# A Meta Analysis of Lumbar Spinal Fusion Surgery Using Bone Morphogenetic Proteins and Autologous Iliac Crest Bone Graft

**DOI:** 10.1371/journal.pone.0097049

**Published:** 2014-06-02

**Authors:** Haifei Zhang, Feng Wang, Lin Ding, Zhiyu Zhang, Deri Sun, Xinmin Feng, Jiuli An, Yue Zhu

**Affiliations:** 1 Department of Orthopedics, The fourth Affiliated Hospital, China Medical University, Shenyang, China; 2 China Medical University Computer Center, Shenyang, China; 3 Department of Orthopedics, The first Affiliated Hospital, China Medical University, Shenyang, China; Georgia Regents University, College of Dental Medicine, United States of America

## Abstract

**Background:**

Bone morphogenetic protein (BMPs) as a substitute for iliac crest bone graft (ICBG) has been increasingly widely used in lumbar fusion. The purpose of this study is to systematically compare the effectiveness and safety of fusion with BMPs for the treatment of lumbar disease.

**Methods:**

Cochrane review methods were used to analyze all relevant randomized controlled trials (RCTs) published up to nov 2013.

**Results:**

19 RCTs (1,852 patients) met the inclusion criteria. BMPs group significantly increased fusion rate (RR: 1.13; 95% CI 1.05–1.23, P = 0.001), while there was no statistical difference in overall success of clinical outcomes (RR: 1.04; 95% CI 0.95–1.13, P = 0.38) and complications (RR: 0.96; 95% CI 0.85–1.09, p = 0.54). A significant reduction of the reoperation rate was found in BMPs group (RR: 0.57; 95% CI 0.42–0.77, p = 0.0002). Significant difference was found in the operating time (MD−0.32; 95% CI−0.55, −0.08; P = 0.009), but no significant difference was found in the blood loss, the hospital stay, patient satisfaction, and work status.

**Conclusion:**

Compared with ICBG, BMPs in lumbar fusion can increase the fusion rate, while reduce the reoperation rate and operating time. However, it doesn’t increase the complication rate, the amount of blood loss and hospital stay. No significant difference was found in the overall success of clinical outcome of the two groups.

## Introduction

Autogenous iliac crest bone graft (ICBG) is considered the gold standard graft material for lumbar fusion, but there are several serious shortcomings in performing lumbar arthrodesis with ICBG, including donor-site morbidity and relatively high frequency of nonunion. Additionally, the amount and quantity of autogenous bone graft are limited and may be insufficient, particularly in arthrodesis over multiple segments [1]. In an effort to decrease the reliance on autograft, bone morphogenetic protein (BMPs) which Urist first described in 1965 had been utilized to supplement or replace the bone graft in spinal fusion surgery, but mass production of this molecule became feasible after the sequencing of multiple BMP genes in the 1990s [2], [3]. Human BMP is now produced on a large scale using recombinant techniques. Since the FDA, investigational device exemption for rhBMP-2 in 1996 and for rhBMP-7 in 2001, both BMPs have been under clinical investigation in various trials. So, we conducted this meta-analysis to assess the effectiveness and safety of BMPs compared with ICBG in lumbar fusion.

## Materials and Methods

### Literature Search

A protocol was developed in advance of conducting this meta-analysis following the Cochrane Back Review Group guidelines [6]. Updating to November 2013, the relevant RCTs in all languages were identified through computer and other research methods. The sources of computer searching include PubMed, The Cochrane Central Register of Controlled Trials (CENTRAL), Ovid MEDLINE and EMBASE, CINAHL, the China Biological Medicine Database (CBM), International Clinical Trials Registry Platform (ICTRP),Current Controlled Trials,ClinicalTrials.gov. Other searching methods include screening references listed in relevant systematic reviews and identified RCTs, and searching abstracts of relevant meetings, and personal communication with content experts in the field and with authors of identified RCTs. Key words that have been used for researching are lumbar degenerative disease (LDD), low back pain, lumbar fusion, bone morphogenetic protein-2**,** bone morphogenetic protein-7, osteogenic protein-1, and randomized controlled trial.

### Study Eligibility Criteria

All RCTs comparing the BMPs to ICBG for the treatment of LDD were identified in this study. Patients older than eighteen years of age with systematic LDD were included in the review. Articles were regarded eligible if they met the following inclusion criteria: the target population comprised adult patients suffering from degenerative conditions of the lumbar spine requiring fusion; the main intervention was lumbar fusion using BMPs as a substitute to ICBG; each potentially eligible study included a comparison group of patients in whom ICBG was used as the only biologic enhancement of the fusion process. Articles were excluded if they reported on patient populations with any of the following characteristics: spinal deformities in adolescents, fractures of the spinal column, spondylolisthesis classified as higher than Meyerding Grade 2, a regular postoperative regimen of pharmaceutical agents that potentially could interfere with fusion (such as steroids or chemotherapy agents).

The trial selection process was based on a first phase of title and abstract screening followed by a second phase of eligibility evaluation from the full-text format. Both actions were performed by two reviewers and checked by the principal reviewer. The observed percentage agreement between the reviewers for the assessment of inclusion was calculated using the κ test [4], [5]. Disagreements were resolved by discussion.

### Risk of Bias Assessment and Evaluation of Validity

The risk of bias (RoB) and methodological quality was assessed in duplicate using the 12 criteria recommended by the Cochrane Back Review Group and evaluated independently by two review investigators [6], [7]. A study with a low RoB was defined as one fulfilling six or more of the criteria items, which is supported by empirical evidence, and with no fatal flaw, which is defined as those studies with (1) a dropout rate greater than 50% at the first and subsequent follow-up measurements or (2) statistically and clinically relevant important baseline differences for one or more primary outcomes indicating unsuccessful randomization. The quality of the evidence related to the estimation of lumbar fusion with BMPs and ICBG followed the suggestions of the GRADE Working Group by adopting the use of GradePro (version 3.6).

### Data Extraction

The data were extracted from included reports independently by two reviewers, and further discussions were done to deal with the disagreements. The data extracted included the following categories: the participant characteristics, the number of participants, and the loss to follow-up; study characteristics; intervention details; the primary and the secondary outcomes. The primary outcomes included: (1) the solid fusion rate, (2) clinical outcomes, (3) complications, and (4) the reoperation rate. The secondary outcomes included: (1) the operation time and blood loss, and hospital stay, (2) patient satisfaction with the treatment, (3) work status and return to work rate.

### Assessment of Heterogeneity

Heterogeneity was explored in two manners, informally by vision (eye-ball test) and formally tested by the Q-test (chi-square) and I^2^; however, the decision regarding heterogeneity was dependent on I^2^. Substantial heterogeneity is defined as ≥50%, and where necessary, the effect of the interventions is described if the results are too heterogeneous.

### Assessment of Clinical Relevance

Two reviewers independently assessed the clinical relevance of included studies according to 5 questions that were recommended by the Cochrane Back Review Group [6]. Each question was scored positive (+) if the clinical relevance item was met, negative (−) if the item was not met, and unclear (?) if data were not available to answer the question. A 20% improvement in pain scores and a 10% improvement in functioning outcomes were considered to be clinically important.

### Measures of Treatment Effect

Attempts were made to statistically pool the data of homogeneous studies in order to obtain the primary and the secondary outcomes. The results were expressed in terms of risk ratio (RR) and a 95% confidence interval (95% CI) for dichotomous outcomes, and in terms of mean difference (MD) and 95% CI for continuous outcomes. When the same continuous outcomes are measured in different scales, standardized mean difference (SMD) and 95% CI are calculated. If in some studies outcomes are shown as dichotomous data while in the other studies expressed as continuous data, RRs would be expressed as SMD to allow dichotomous and continuous data to be pooled together [6]. Collected data were checked and entered into the computer by the two reviewers. A random-effects model was used in this meta-analysis [6], [8]. We performed a sensitivity analysis for the measured effects omitting studies with low methodological quality which may largely influence the clinical results. Funnel plot and statistic tests (Egger’s test and Begg’s test) were used to explore potential publication bias [9]–[11]. To assess the stability in the overall result if publication bias existed, we corrected the summary results by the trim and fill method [12], [13]. RevMan software (vesion5.1.0) and the R project (vesion3.0.1) were used for data analysis.

## Results

### Search Results

**Figure 1:**
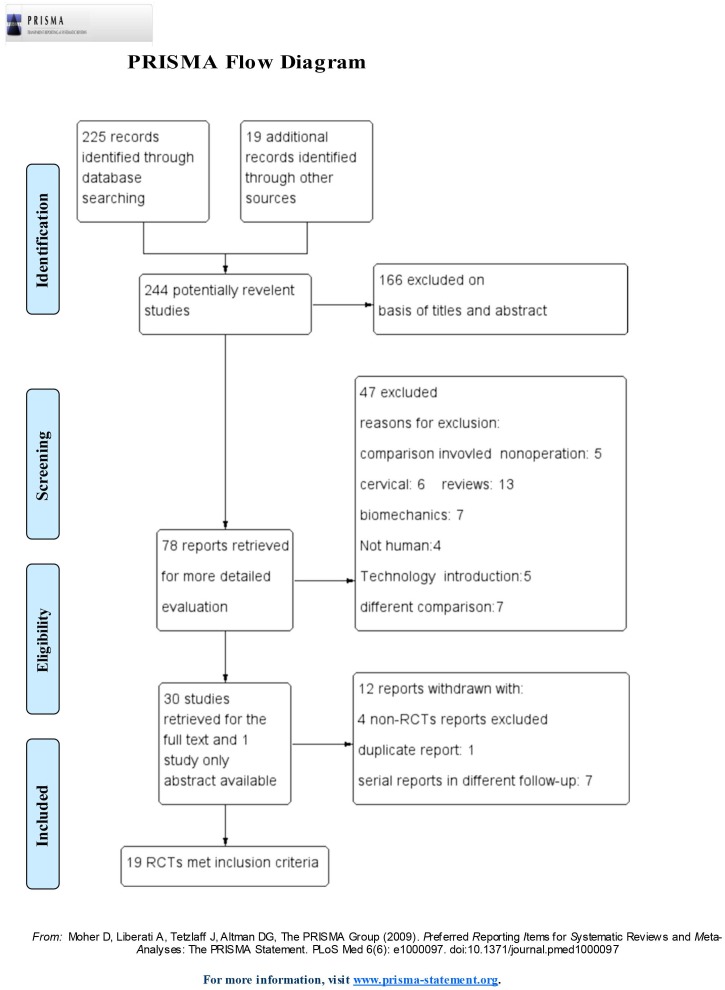
PRISMA flow diagram.

The primary search identified 244 records, and 166 publications were immediately excluded based on titles and abstracts. From the potentially relevant 78 publications, 59 were omitted according to the inclusion criteria. Finally, 19 trials [14]–[32] were included in the meta-analysis (Fig. 1). The κ statistic for interrate agreement in terms of study eligibility was 0.81.

19 RCTs (15 bmp-2, 4 bmp-7) involving 1852 patients were deemed eligible for inclusion, with individual sample sizes ranging from 14 to 463 patients. All the included studies have definite inclusion/exclusion criteria. Those studies recruited patients with a variety of spinal disorders, and surgical treatment involved ALIF fusion, PLIF fusion or PLF fusion (instrumented OR uninstrumented). In one study [15], there were two treatment groups (rhBMP-2/TSRH group and rhBMP-2 only group) and one control group (ICBG/TSRH group). To avoid heterogeneity, rhBMP-2 only group was omitted from this Meta analysis. Characteristics of included studies were presented in Table 1.

**Table 1:** Overview of included trials.

1^st^Author, publication year	Country conducted	Preoperative diagnosis	Comparisons	Sample sizeT/C	Female (%)	Mean age(year)T/C	Follow-up (month)	Follow-up rate(%)
Boden 2000 [14]	United States	Single-level lumbar DDDSpondylolisthesis ≤ grade 1	rhBMP-2/ACS vs. ICBG (ALIF)	11/3	50	42.5/40.2	24	100
Boden 2002 [15]	United States	Single-level lumbar DDDSpondylolisthesis ≤ grade 1	rhBMP-2/BCP vs. ICBG (PLF with/without TSRH)	11/5	68.8	57.6/52.9	17	100
Burkus Gornet 2002[16]	United States	Single-level lumbar DDD	rhBMP-2/ACS vs. ICBG.(ALIF)	143/136	47.7	43.3/42.3	24	91.7
Burkus Transfeldt 2002 [17]	United States	Single-level lumbar DDD	rhBMP-2(InFUSE) vs. ICBG.(ALIF)	24/22	60.9	41.5/45.6	24	95.7
Johnsson 2002 [18]	Sweden	L5 spondylolysis and vertebral slip ≤50%,	BMP-7 vs. ICBG.(uninstrumented PLF)	10/10	60	42.9/40.4	12	100
Burkus 2003 [19]	United States	degenerative lumbar spondylosis	rhBMP-2/ACS vs. ICBG.(ALIF)	22/20	47.6	41.7/44.2	24	100
Assiri 2004 [20]	Canada	DDD	rhBMP-2 vs. ICBG. (PLF)	8/7	NR	NR	24	NR
Haid 2004 [21]	United States	Single-level lumbar DDDSpondylolisthesis ≤ grade 1	rhBMP-2/ACS vs. ICBG.(PLIF)	34/33	52.2	46.3/46.1	24	94
Glassman 2005 [22]	United States	Single-level lumbar DDDSpondylolisthesis ≤ grade 1	rhBMP-2/CRM vs. ICBG.(PLF)	38/36	59.5	53/53	12	97
Vaccaro 2005 [23]	United States	single-level degenerativespondylolisthesis and stenosis	BMP-7 vs. ICBG.(uninstrumented PLF)	24/12	55.6	63/66	24	88.9
Burkus 2005 [24]	United States	Single-level lumbar DDD	rhBMP-2/ACS vs. ICBG.(ALIF)	79/52	61.1	40.2/43.6	24	99.2
Dimar 2006 [25]	United States	Single-level lumbar DDDSpondylolisthesis ≤ grade 1	rhBMP-2/CRM vs. ICBG.(PLF)	53/45	57.1	50.9/52.7	24	100
Kanayama 2006 [26]	Japan	degenerativespondylolisthesis with stenosis	BMP-7 vs. ICBG.(PLF)	9/10	42.1	70.3/58.7	13–16	100
Glassman 2008 [27]	United States	Single/multilevel lumbar DDD; spondylolisthesis; stenosis;adjacent level fusion	rhBMP-2/ACS vs. ICBG.(PLF)	50/52	68.6	69.9/69.2	24	94.3
Vaccaro 2008 [28]	United States	single-level degenerativespondylolisthesis and stenosis	BMP-7 vs. ICBG.(uninstrumented PLF)	208/87	66.9	68/69	>48	80
Dimar 2009 [29]	United States	Single-level lumbar DDDSpondylolisthesis ≤ grade 1	rhBMP-2 matrix vs. ICBG.(PLIF)	239/224	56.2	53.2/52.3	24	89
Dawson 2009 [30]	United States	Single-level lumbar DDDSpondylolisthesis ≤ grade 1	rhBMP-2/ACS vs. ICBG.(PLIF)	25/21	58.7	55.9/56.9	24	87
Delawi 2010 [31]	Netherlands	Spondylolisthesis ≤ grade 2	BMP-7 vs. ICBG.(PLF)	18/16	52.9	53/55	12	94.4
Michielsen 2013 [32]	Belgium	Single-level lumbar DDD	rhBMP-2/ACS vs. ICBG.(PLIF)	19/19	57.8	43.2/42.2	12	100

ACS: absorbable collagen sponge carrier; ICBG: autogenous iliac crest bone graft;

BCP: biphasic calcium phosphate; CRM: compression resistant matrix; DDD: degenerative disc disease; NR: no report.

### Methodological Quality of the Included Studies

**Figure 2:**
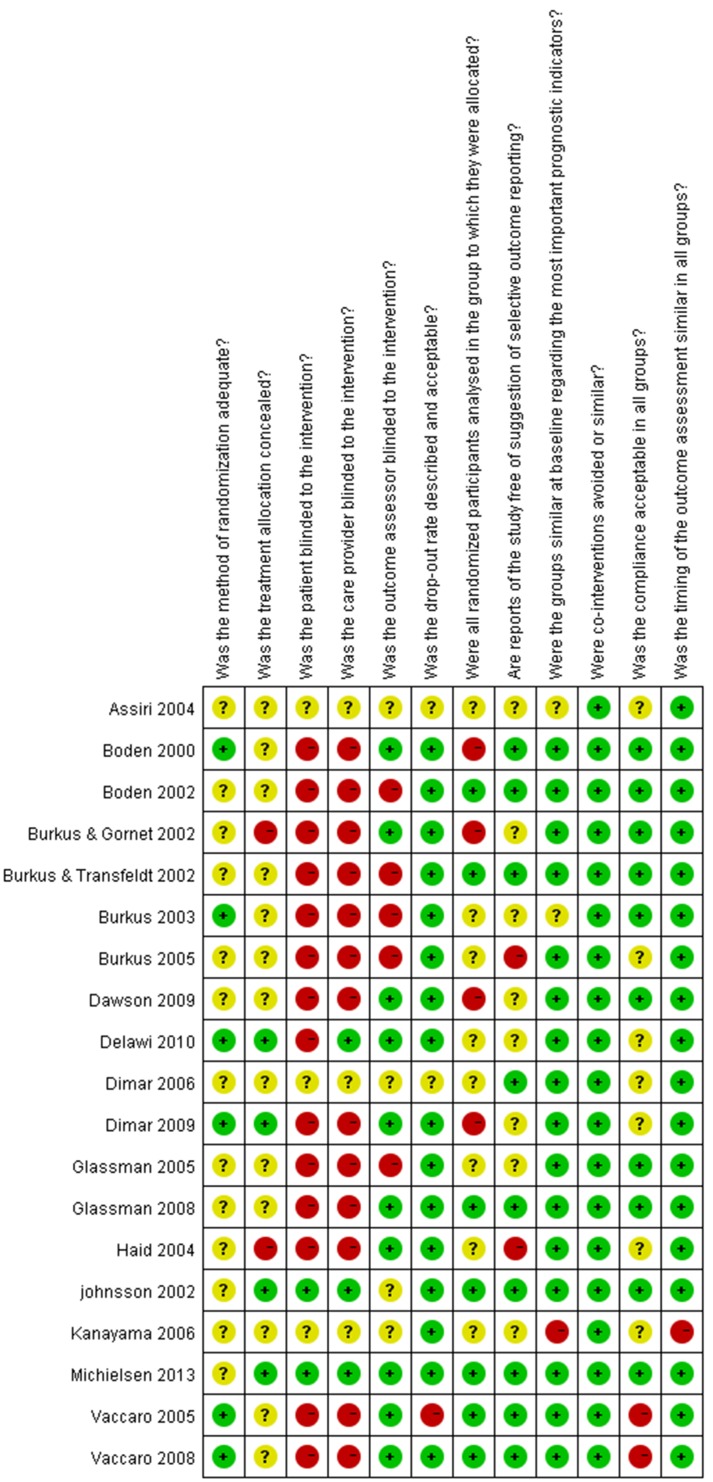
Risk of bias summary.

The results of the RoB for the individual studies are summarized in Figure 2. In total, 12 of the 19 trials met the criteria for a low RoB [14]–[18], [23], [27]–[32]. 6 studies have adequate methods of randomization [14], [19], [23], [28], [29], [31], and only two studies use both an adequate sequence generation and allocation procedure [29], [31]. In 9 studies, both randomization and allocation were unclear [15], [17], [20], [22], [24], [25]–[27], [30].

No reliable studies attempted to blind patients or surgeon because this was impossible due to the nature of the surgery. The lack of blinding was compensated by using blinded observers to assess the fusion outcome in 10 studies [14], [16], [21], [23], [27]–[32]. To prevent any potential bias in surgical technique between the treatment groups, 3 studies [18], [31], [32] revealed the randomization at the end of the surgery, just before the graft was needed. So, we considered those studies as blinded care provider.

Most of the studies provided an adequate overview of withdrawals or dropouts and were able to keep these to a minimum for the subsequent follow-up measurements, although only Vaccaro and Burkus conducted long-term follow-up [24], [28].

Published or registered protocols were unavailable for all studies, though we conducted a comprehensive search. In the absence of these, it was difficult for us to decide whether outcomes were measured, but not reported because they were found to be insignificant or unfavorable. Therefore, only eight studies reporting all four primary outcomes (i.e., the solid fusion rate, clinical outcomes, complications, and the reoperation rate) were considered to have fulfilled this criterion [14], [15], [17], [23], [25], [27], [28], [32].

The quality of the overall body of evidence for each individual outcome was addressed and summarized through the GRADE system (Table 2). The assessment of the solid fusion rate as a primary outcome was rated as moderate quality, in view of high risk of bias in seven trial designs and implementation. As other primary outcomes, overall success and reoperation rate were also rated as low quality because of imprecision and/or reporting bias, and complications were rated as moderate quality on account of imprecision. However, the secondary outcomes were rated as moderate quality, low quality, or very low quality, results from assessing pooled events of patient satisfaction, surgical conditions and work status respectively.

**Table 2:** GRADE profile for the quality of evidence related to the assessment for Lumbar Spine using BMPs and ICBG.

Quality assessment							No of patients		Effect		Quality	Importance
				
No of studies	Design	Risk of bias	Inconsistency	Indirectness	Imprecision	Other considerations	BMPs	ICBG	Relative(95% CI)	Absolute		
**solid fusion rate (follow-up mean 24 months)**												
17	randomised trials	serious^1^	no serious inconsistency	no serious indirectness	no serious imprecision	none	547/610 (89.7%)	410/523 (78.4%)	RR 1.13 (1.05 to 1.23)	102 more per 1000 (from 39 more to 180 more)	⊕⊕⊕○	CRITICAL
								70.6%		92 more per 1000 (from 35 more to 162 more)	MODERATE	
**overall success of clinical outcomes (follow-up mean 24 months)**												
8	randomised trials	no serious risk of bias	no serious inconsistency	no serious indirectness	serious^3^	reporting bias^2^	339/431 (78.7%)	199/265 (75.1%)	RR 1.04 (0.95 to 1.13)	30 more per 1000 (from 38 fewer to 98 more)	⊕⊕○○	CRITICAL
								70%		28 more per 1000 (from 35 fewer to 91 more)	LOW	
**Complications (follow-up mean 24 months)**												
9	randomised trials	no serious risk of bias	no serious inconsistency	no serious indirectness	serious^3^	none	112/605 (18.5%)	87/444 (19.6%)	RR 0.96 (0.85 to 1.09)	8 fewer per 1000 (from 29 fewer to 18 more)	⊕⊕⊕○	CRITICAL
								23.1%		9 fewer per 1000 (from 35 fewer to 21 more)	MODERATE	
**Reoperation rate (follow-up mean 24 months)**												
14	randomised trials	Serious^4^	no serious inconsistency	no serious indirectness	Serious^5^	none	72/1004(7.2%)	100/766(13.1%)	RR 0.57 (0.42 to 0.77)	56 fewer per 1000 (from 30 fewer to 76 fewer)	⊕⊕○○	CRITICAL
								10.2%		44 fewer per 1000 (from 23 fewer to 59 fewer)	LOW	
**operative time (follow-up mean 24 months; Better indicated by lower values)**												
9	randomised trials	no serious risk of bias	very serious^6^	no serious indirectness	serious^3^	reporting bias^2^	435	388		MD 0.32 lower (0.55 to 0.08 lower)	⊕○○○	IMPORTANT
											?VERY LOW	
**Blood loss (follow-up mean 24 months; Better indicated by lower values)**												
8	randomised trials	no serious risk of bias	very serious^7^	no serious indirectness	Serious^3^	reporting bias^2^	411	376		MD 50.24 lower (117.38 lower to 16.9 higher)	⊕○○○	IMPORTANT
											?VERY LOW	
**Hospital stay (follow-up mean 24 months; Better indicated by lower values)**												
7	randomised trials	no serious risk of bias	Serious^8^	no serious indirectness	serious^3^	none	377	332		MD 0.56 lower (1.12 to 0.01 lower)	⊕⊕○○	IMPORTANT
											LOW	
**Patient satisfaction rate (follow-up mean 24 months)**												
4	randomised trials	no serious risk of bias	no serious inconsistency	no serious indirectness	Serious^3^	none	147/186 (79%)	125/165 (75.8%)	RR 1.06 (0.86 to 1.32)	45 more per 1000 (from 106 fewer to 242 more)	⊕⊕⊕○	IMPORTANT
								70%		42 more per 1000 (from 98 fewer to 224 more)	MODERATE	
**Return-to-work status (follow-up mean 24 months)**												
2	randomised trials	serious^9^	serious^10^	no serious indirectness	very serious^11^	none	22/23 (95.7%)	24/27 (88.9%)	RR 1.1 (0.69 to 1.76)	89 more per 1000 (from 276 fewer to 676 more)	⊕⊕○○	IMPORTANT
								83.3%		83 more per 1000 (from 258 fewer to 633 more)	VERY LOW	
**Work status (follow-up mean 24 months)**												
7	randomised trials	no serious risk of bias	no serious inconsistency	no serious indirectness	Serious^3^	none	229/471 (48.6%)	197/416 (47.4%)	RR 1.05 (0.85 to 1.3)	24 more per 1000 (from 71 fewer to 142 more)	⊕⊕⊕○	IMPORTANT
								42.4%		21 more per 1000 (from 64 fewer to 127 more)	MODERATE	

1 seven studies with a high risk of bias;

2 Asymmetry in funnel plot;

3 less than 75% of the studies present data that can be included in a meta-analysis;

4 five studies with a high risk of bias;

5 RR = 0.56;

6 I^2^ = 79%;

7 I^2^ = 77%;

8 I^2^ = 70%;

9 one study including more than 50% patients has a high risk of bias;

10 I^2^ = 70%;

11 only two studies including total 50 patients present data that can be included in a meta-analysis.

### Clinical Relevance

Clinical relevance of included studies was presented in Table 3. The κ statistic for interrate agreement in terms of study eligibility was 0.83. Consensus was reached on all scorings after discussion. The reviewers considered the likely treatment benefits to be worth the potential harms in 13 studies [14], [16], [17], [21]–[24], [25], [27]–[31], and the size of the effect was considered to be clinically important in eight studies [14], [15], [17], [22]–[24], [30], [32], and all clinically relevant outcomes were considered to be measured and reported in nine studies [14], [15], [17], [23], [25], [27], [28], [31], [32]. Most of the included trials described the interventions and treatment settings well enough to enable clinicians to replicate the treatment in clinical practice.

**Table 3:** Clinical relevance.

		Boden 2000	Boden 2002	Burkus & Gornet 2002	Burkus& Transfeldt2002	johnsson 2002	Burkus 2003	Assiri 2004	Haid2004	Burkus2005	Glassman 2005	Vaccaro 2005	Dimar2006	Kanayama 2006	Glassman 2008	Vaccaro 2008	Dawson 2009	Dimar 2009	Delawi 2010	Michielsen 2013
1	Are the patients described in detail so that you can decide whetherthey are comparable to those that you see in your practice?	+	+	+	+	+	+	?	+	+	+	?	+	+	+	+	+	+	+	+
2	Are the interventions and treatment settings described well enoughso that you can provide the same for your patients?	+	+	+	+	+	+	?	+	+	+	+	+	+	+	+	+	+	+	+
3	Were all clinically relevant outcomes measured and reported?	+	+	?	+	?	−	?	_	?	+	?	?	+	+	?	+	+	+	+
4	Is the size of the effect clinically important?	+	+	−	+	?	−	?	+	+	+	−	−	−	−	+	−	−	−	+
5	Are the likely treatment benefits worth the potential harms?	+	?	+	+	−	+	?	+	+	+	+	?	+	+	+	+	+	+	−

### Quantitative Data Synthesis

**Figure 3:**
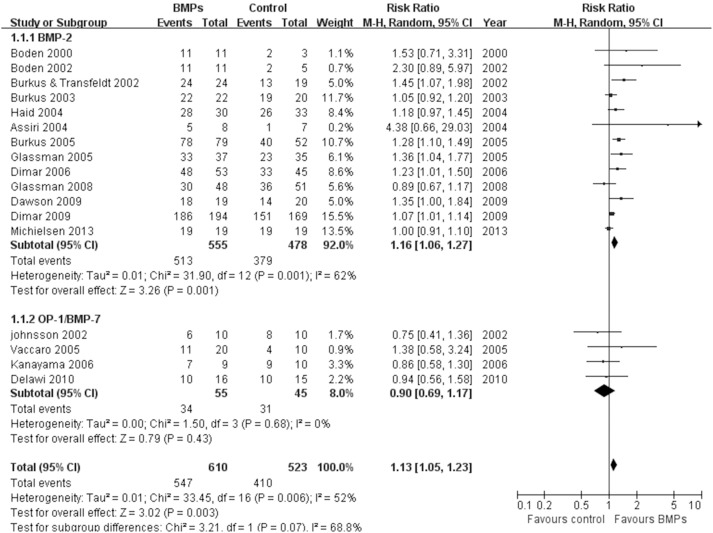
Forest plot-fusion rate.

**Figure 4:**
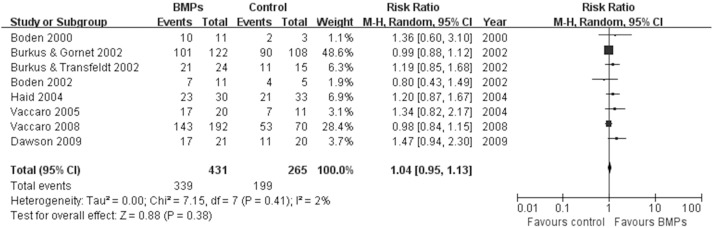
Forest plot- overall clinical success.

**Figure 5:**
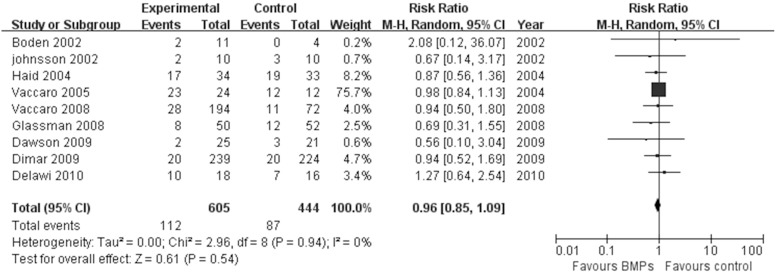
Forest plot- complications.

**Figure 6:**
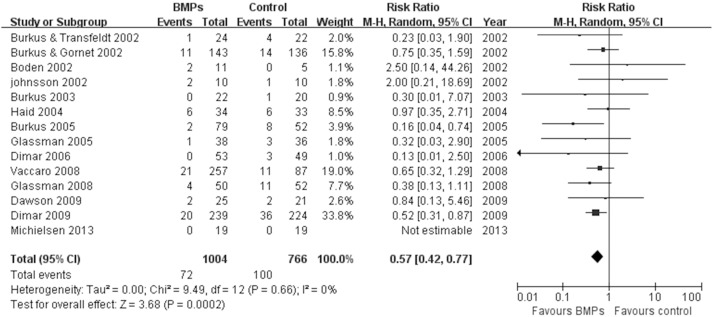
Forest plot- reoperation rate.

17 studies [14], [15], [17]–[27], [29]–[32] assessed the fusion rate between BMPs and ICBG (610 participants with BMPs and 523 with ICBG), significant differences were found in comparisons (RR: 1.13; 95% CI 1.05–1.23; *P* = 0.003). Heterogeneity was obvious during follow-up 24 months, I^2^ = 52%. We also have a subgroup analysis. Similar results were obtained by pooled only BMP-2 studies (RR: 1.16; 95% CI 1.06–1.27; *P* = 0.001), by contrast, pooled BMP-7 studies have different results (RR: 0.90; 95% CI 0.69–1.17; *P* = 0.43). Heterogeneity were moderate or absent in bmp-2 subgroup and bmp-7 subgroup, respectively (BMP-2: I^2^ = 62%; BMP-7: I^2^ = 0; Fig. 3). Data for overall success of clinical outcomes were available in 8 studies (431 participants with BMPs and 265 with ICBG) [14]–[17], [21], [23], [28], [30].No significant difference was found between two groups (RR: 1.04; 95% CI 0.95–1.13; *P* = 0.38). There was no significant heterogeneity between trials (I^2^ = 2%; Fig. 4). With regard to complications, we pooled data of 9 trials [15], [21], [23], [27]–[31] about the frequency of adverse reactions (605 participants with BMPs and 444 with ICBG). The frequency of adverse events or complications was similar in both groups (RR = 0.96; 95% CI 0.85–1.09; p = 0.54). There was no heterogeneity between the studies (I^2^ = 0%; Fig. 5). The reoperation rate of the BMPs group and the ICBG group was available in 14 studies (1004 participants with BMPs and 766 with ICBG) [15]–[19], [21], [22], [24], [25], [27]–[30], [32]. A significant reduction of the reoperation rate was found in subjects receiving lumbar fusion with BMPs (RR = 0.57; 95% CI 0.42–0.77; p = 0.0002), and no substantial heterogeneity was found (I^2^ = 0%; Fig. 6).

**Figure 7:**
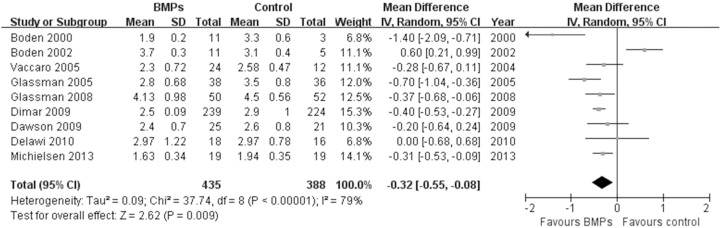
Forest plot- operating time.

**Figure 8:**
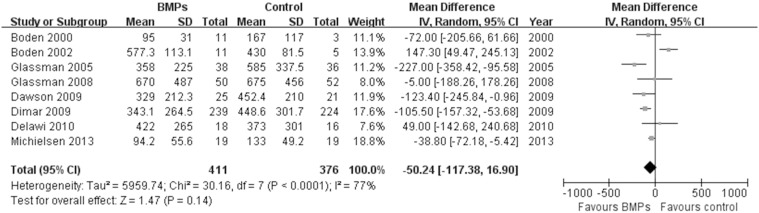
Forest plot- blood loss.

**Figure 9:**
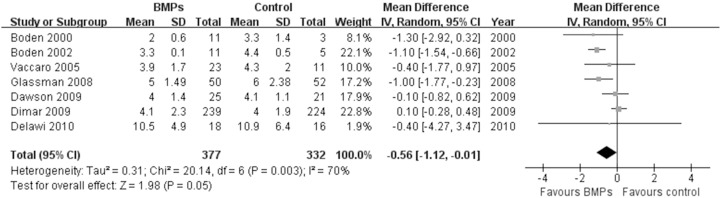
Forest plot- hospital stay.

**Figure 10:**
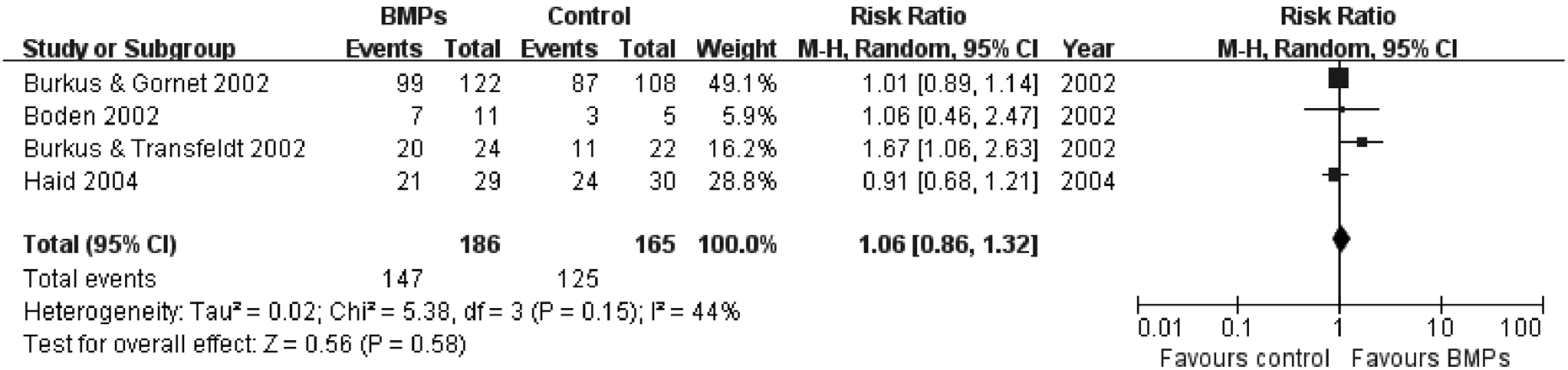
Forest plot- patient satisfaction.

**Figure 11:**
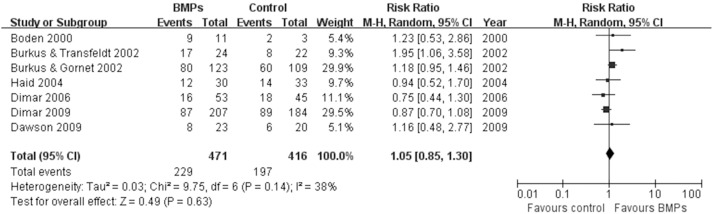
Forest plot- work status.

**Figure 12:**
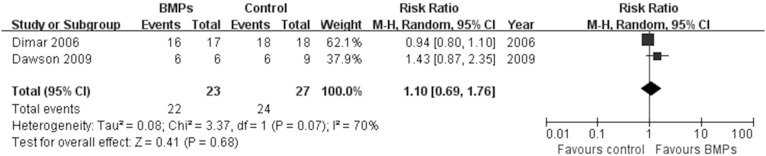
Forest plot- return to work status.

In the secondary outcomes, significant difference was found in the operating time between two groups in 9 trials [14], [15], [22], [23], [27], [29]–[32], (MD−0.32; 95% CI−0.55, −0.08; P = 0.009), it had obviously heterogeneity (I^2^ = 79%; Fig. 7). However, no significant difference was found in the Blood loss between two groups in 8 trials [14], [15], [22], [25], [27], [30]–[32], (MD−50.24; 95% CI−117.38, 16.90; P = 0.14), it also had obviously heterogeneity (I^2^ = 77%; Fig. 8). No significant difference was found in the hospital stay in 7 trials [9], [10], [15], [16], [18], [20], [23] (MD−0.56; 95% CI: −1.12, −0.01; *P* = 0.05). It also had obviously heterogeneity (I^2^ = 70%; Fig. 9). Patient satisfaction was available from 4 included studies [15]–[17], [21]. The pooled result showed no significant difference in the BMPs group in comparison to the ICBG group (RR = 1.06; 95% CI 0.86–1.32; p = 0.58), and a moderate heterogeneity was found (I^2^ = 44%; Fig. 10). The data of patients’ work status were available in 7 studies [9], [11], [12], [15], [17], [18], [21] at 24 months follow up. No significant difference was found between two groups (RR = 1.05; 95% CI 0.85–1.30; *P* = 0.63). There was no significant heterogeneity between trials (I^2^ = 38%; Fig. 11). No significant difference was found about return-to-work status in 2 trials [15], [17] (RR 1.10; 95% CI 0.69–1.76; *P* = 0.68). It also had obviously heterogeneity (I^2^ = 70%; Fig. 12).

### Qualitative Data Synthesis

#### Donor pain

Burkus et al [16] found that all the control patients experienced donor site hip pain after surgery. The mean pain score was 12.7 points out of 20 points immediately after surgery, however, at 24 months after surgery pain scores averaged 1.8 points, and 32% patients still experienced pain. In his other study [17], the mean graft-site pain was highest (11.3) after surgery, but it was reduced to 2.2 at 24 months. He also reported 46.5% of the control group patients had persistent pain for 24 months after surgery in the subsequent study in 2005[24]. Haid et al [21] found similar result that the highest levels of pain were noted immediately after surgery with a mean score of 11.6 points, however, at 24 months after surgery, 60% of the control patients still experienced pain, and the graft site pain scores averaged 5.5 points. Dimar et al [25] measured donor site pain utilizing hip pain scores. The mean score after surgery was 11.6, which improved to 7.6 at 24 months after surgery. Vaccaro et al [28] reported 45% of the control group patients had persisted pain for 24 months after surgery, and 35% of the control group patients had persisted mild/moderate pain for 36 months after surgery. Donor site pain was persistent and decreased slowly over time, reported as 1.2 on the VAS (scale of 1–10, 10 being most severe) at 24 months, and 1.1 at 36 months. Dimar et al [29] measured donor site pain using donor-site pain scores. The mean score after discharge was 11.3, which improved to 5.1 at 24 months after surgery,and 60% of the control group patients had persistent pain. Dalewi et al [31] reported that the average donor site pain at 1-year follow-up was graded as 2.7+/−2.8 using the VAS. No complication directly related to the bone graft harvesting procedure occurred.

#### Antibody formation

Six studies assessed antibody responses to BMPs or bovine collagen after surgery. Boden et al [14] did not detect an elevated antibody response to rhBMP-2 in any of the 11 patients, although 3 patients (27%) developed antibodies to bovine type I collagen. No complications were associated with these antibody responses. In the subsequent study, they reported a transient antibody response to rhBMP-2 in 1 of 22 patients (4.5%) and 0% (0/4) in the autograft group 3 months after surgery [15]. In Burkus’s study [16], antibodies to rhBMP-2 were evaluated preoperatively and at 3 months after surgery. The results were similar between the rhBMP-2 and control groups. There appeared to be no negative consequence to positive antibody test results. Similarly, 3 months after PLIF with rhBMP-2, Haid et al [21] found that no patients had an elevated antibody response against rhBMP-2, and 3 of 34 patients had developed antibodies against bovine type I collagen. There were no signs of any negative clinical sequelae in patients who tested positive for antibodies against bovine collagen. Burkus et al [24] did not identify an elevated antibody response to rhBMP-2 in any patients, although seven patients (9%) in the study group and four patients (8%) in the control group had an elevated antibody response to bovine collagen. Vaccaro et al [28] found that 25.6% of patients developed neutralizing anti-OP-1 antibodies at any time during follow-up, although there was no association with this neutralizing activity with any clinical outcomes. Further, no neutralizing anti-bodies were detected in the serum of patients at 24 or 36 month follow-up appointments.

### Sensitivity Analysis

To evaluate whether the studies rated to be with high risk of bias significantly affected our results, we performed a sensitivity analysis. The methodological quality was assessed using the 12 criteria recommended by the CBRG. A study with a low RoB was defined as one fulfilling six or more of the criteria items. Therefore, seven studies [19]–[22], [24]–[26] with a high RoB fulfilling less than six of the 12 criteria items were excluded in sensitive analysis. After excluding these studies, the summary RR of fusion rate at 24 months was 1.09 (95% CI = 0.98–1.21, *P* = 0.13). These were significantly different from previous results.

### Publication Bias

**Figure 13:**
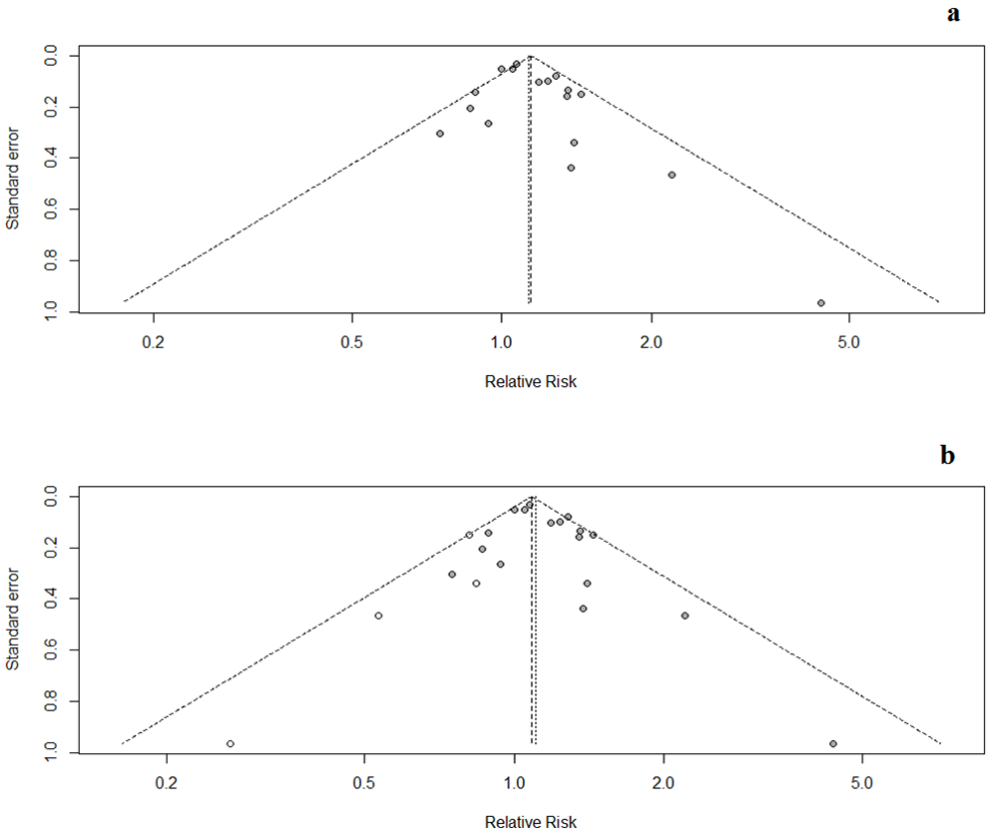
Funnel plot-fusion rate.

The funnel plot of fusion rate at 24 month is presented in Fig. 13a. No evidences of publication bias were found in both Egger’s test (p = 0.12) and Begg’s test (p = 0.56). However, when we corrected for publication bias using the trim and fill method, the effect of BMPs on fusion rate (RR1.10, 95% CI 1.02–1.19) was not clinically different from the uncorrected result (Fig. 13b).

## Discussion

The goal of spine surgery for degenerative spinal disease is oftentimes the attainment of solid union of the degenerated and potentially unstable motion segments [33]. Despite the fact that the use of ICBG is the current standard, the morbidity associated with graft harvest has led surgeons to seek viable alternatives [34]–[38]. BMPs are naturally occurring proteins that stimulate bone healing by a cascade mechanism that results in the differentiation of primitive mesenchymal cells and preosteoblasts into osteoblasts that promote bone formation and, ultimately, healing [39], [40]. Currently, two recombinant human BMPs, rhBMP-2 and rhBMP-7, are available for clinical use. These osteoinductive agents have been approved for lumbar fusions either as autologous bone graft enhancers or even autologous bone graft substitutes. However, serious issues and misconceptions regarding the use of osteoinductive bone graft substitutes have recently been outlined [41]–[43]. So, the purpose of this study is to systematically compare the effectiveness and safety of fusion with BMPs for the treatment of lumbar disease.

This meta-analysis identified 19 RCTs that compared BMPs and ICBG for lumbar fusion. It revealed that there was significant difference in the solid fusion rate and the reoperation rate. Subgroup analysis of the fusion rate stratified by the two types of BMPs yielded different results. Compared with ICBG, the use of BMP-2 can increase solid fusion rate, by contrast, pooled BMP-7 studies do not have similar effects. However, no significant difference was found in overall success of clinical outcomes and complications. The operating time of BMPs group was shorter than the ICBG group, while the amount of blood loss and hospital stay of BMPs group was not significantly higher than the ICBG group. No significant difference was found in patient satisfaction rate and work status.

Ostensibly, these results are consistent with the previous review. Mussano et al [44] showed that the efficacy of BMPs in vertebral lesions was slightly better than that of standard treatment in terms of producing bone consolidation (radiologic outcome relative risk = 1.07; 95% CI 1.01–1.12), along with functionality and pain (clinical outcome relative risk = 1.08; 95% CI 0.97–1.19). Papakostidis et al [45] evaluated the radiographic and clinical effectiveness of BMPs about lumbar posterolateral fusion. They included seven randomized control trials and one prospective comparative study. Their study found that rhBMP-2 was more efficacious to ICBG in promoting fusion, whereas rhBMP-7 appeared equivalent to ICBG in that respect. Patients treated with BMPs had a shorter hospitalization compared with those that were treated with ICBG. BMPs appeared more efficient in instrumented than non-instrumented posterolateral fusions. Agarwal et al [46] conducted a systematic review to compare the efficacy and safety of osteoinductive bone graft substitutes using autografts and allografts in lumbar fusion. RhBMP-2 significantly decreased radiographic nonunion compared to ICBG. Trials of rhBMP-2 suggested reductions in the operating time and surgical blood loss, with less effect on the length of hospital stay. There was no difference in radiographic nonunion with the use of rhBMP-7 when compared with ICBG. Neither rhBMP-2 nor rhBMP-7 demonstrated a significant improvement on the ODI when compared with ICBG. Chen et al [47] conducted a systematic review which including ten randomized controlled trials had a conclusion that the use of rhBMP-2 significantly reduced the risk of fusion failure and the rate of reoperation comparing with ICBG. They also find that there was no statistical difference in clinical improvement on the ODI, although a favorable trend in the rhBMP-2 group was found. Donell et al [48] found that the use of BMP-2 was associated with a statistically significantly higher rate of spinal fusion than the use of ICBG in patients with single-level DDD. There were no significant differences in the ODI and SF-36 score improvements between BMP-2 and control groups. Adverse events reported were similar between two groups, but one study [21] reported significantly more BMP-2 patients with bone formation outside of the space compared with controls. Recently, serial reports based on Yale University Open Data Access-orchestrated project (YODA) showed different results. Fu et al [49] found that rhBMP-2 has no proven clinical advantage over bone graft and may be associated with important harms, making it difficult to identify clear indications for rhBMP-2. Simmonds et al [50], [51] also conducted a individual-participant data meta-analysis (IPDMA).They found that rhBMP-2 increases fusion rates, reduces pain by a clinically insignificant amount, and increases early postsurgical pain compared with ICBG. Evidence of increased cancer incidence is inconclusive.

Imaging was used to assess the status of spinal fusion after surgery. However, imaging evaluation is different from the direct operative exploration [52]. Therefore, the fusion rate from imaging evaluation may not equal the actual fusion rate. Furthermore, imaging methods and the fusion standards were variable. Our Meta analysis also included articles utilized plain radiographs and CT-imaging, or surgical exploration as a method of evaluation of fusion status. Thus, the results of sensitivity analysis were significantly different from previous results, probably because of that the validity of the combined results influenced by the potential variability. In our study, there are some excellent fusion results using rhBMP-2 in spinal fusion, but pooled data of fusion using BMP-7 are not inferior to autograft. The unfavorable results may be due to lesser osteoinductive capacity of BMP-7 compared with BMP-2, the lower effective BMP dose, and a different carrier possibly being inferior to the BMP-2 carrier. Although achieving a solid arthrodesis is a primary aim of spinal fusion surgery, the overall goal is to improve quality of life and mobility. We cannot conduct a quantitative synthesis, because of incomplete data of parameters of clinical outcome. However, we described most studies, which reported pain and functional outcome scores between baseline and follow-up. At all follow-up intervals, there were significant improvements in the clinical outcome measures, including the ODI scores, Short Form-36 scores, and back and leg pain scores in both groups,but no significant differences were found between groups. It would seem that the use of BMPs is of no detriment in terms of improvements in functional outcomes.

The purpose of this meta analysis was to evaluate the effectiveness and, more importantly, safety of BMPs compared with ICBG in lumbar fusion. Though some reports lack valid data, a quantitative analysis of complications was conducted, which had no significant difference between BMPs and ICBG group. In a systematic review focusing on the safety of BMP-2, Morz et al [53] determined that multiple complications are associated after the use of rhBMP-2 in both cervical and lumbar spine fusion surgery. There was a mean incidence of 44%, 25%, and 27% of resorption, subsidence, and interbody cage migration reported for lumbar spine interbody fusion surgery although reoperation or long-term detrimental effect was rare. Carragee et al [43] concluded that original industry-sponsored trials underestimated BMP-related adverse events, and they thought the risk of adverse events should be considered in the context of demonstrated benefits. Evidence from YODA serial studies [49]–[51] also indicated that there appears to have been an increased risk of uncommon and serious complications with the use of BMPs in lumbar fusion. Therefore, in sum, it is difficult for us to determine the nature, range, and frequency of adverse events associated with BMPs.

Our review has limitations. First, the search was restricted to reports of RCTs published in peer-reviewed journals, excluding other sources of biomedical literature, which could have possibly collected more studies related to the topic. In such a case, studies with positive or statistically significant results would be expected to be over represented in our review; such studies are more likely to be published, particularly in the English language. So we used the funnel plot as a tool to investigate how much our results were potentially influenced by publication bias. Second, the validity of our results is limited by the low quality of the studies included, such as double-blinding was unattainable for most of the trials, that may decrease the strength of conclusions drawn from the meta-analysis. Third, there is the potential for bias because device manufacturers sponsored several studies and some authors reported conflicts of interest. However, there were several improvements in this meta-analysis compare with previous systematic reviews. First, this review is the most current report on the topic and includes the recently published trials. It adopted more strict inclusion criteria. Quasi-RCT and non-RCTs were strictly excluded in this study in order to guarantee the reliability of results. Second, we pooled the data of comparable parameters regarding complications to reduce the bias of the descriptive analysis. Third, we also did an additional qualitative data synthesis of donor pain and antibody responses to BMPs. Fourth, the quality of the overall body of evidence for each individual outcome was addressed and summarized through the GRADE system, that provided a better guideline for the clinical practice.

## Conclusion

In summary, our review adds to the evidence concerning the use of BMPs for lumbar fusion. Various RCT studies conclude that the use of BMPs can increase the fusion rate slightly, while decrease the reoperation rate and operating time. There was no significant difference in the overall success of clinical outcome, the complication rate, the amount of blood loss and hospital stay between the two groups. The use of BMPs prevents graft site related adverse effects. No complications were associated with antibody responses. From the limited evidence, BMPs does not show significant superiority for the treatment of LDD compared with ICBG. To assess the effectiveness and safety of lumbar fusion with BMPs, more high-quality RCTs with long term outcomes are needed.

## Supporting Information

Checklist S1 **PRISMA 2009 Checklist.**
(DOC)

Search strategies S1 (DOC)

List S1 **List of included-excluded studies.**
(DOC)
