# Correction: A Meta Analysis of Lumbar Spinal Fusion Surgery Using Bone Morphogenetic Proteins and Autologous Iliac Crest Bone Graft

**DOI:** 10.1371/journal.pone.0116460

**Published:** 2014-12-19

**Authors:** The PLOS ONE Staff

The first author, Haifei Zhang, is missing an affiliation. Haifei Zhang should also be affiliated with: Department of Orthopedics, The first Affiliated Hospital, China Medical University, Shenyang, China
