# Supplementary material for: A Meta Analysis of Lumbar Spinal Fusion Surgery Using Bone Morphogenetic Proteins and Autologous Iliac Crest Bone Graft
Source: PLoS One. 2014 Jun 2;9(6):e97049. doi: 10.1371/journal.pone.0097049 (PMC4041715; doi:10.1371/journal.pone.0097049)
Supplement: Search strategies S1 — (DOC) [file pone.0097049.s002.doc]

**Search strategies of the major electronic databases**

*Medline search strategy*

1. ((bone morphogen$ or osteogen$ or osteoinduct$) adj (protein$ or factor$ or polypeptide$ or poly-peptide$)).ti,ab.

2. (BMP2 or BMP-2 or rhBMP2 or rhBMP-2 or rh-BMP2 or rh-BMP-2).ti,ab.

3. (BMP7 or BMP-7 or rhBMP7 or rhBMP-7 or rh-BMP7 or rh-BMP-7).ti,ab.

4. (OP1 or OP-1 or rhOP1 or rhOP-1 or rh-OP1 or rh-OP-1).ti,ab.

5. (InductOs or InFuse).ti,ab.

6. exp Bone Morphogenetic Proteins/

7. exp Spinal Diseases/

8. exp Spinal Fusion/

9. exp Spine/

10. (spine or spinal or thoracic or lumbar or cervical).ti,ab.

11. (lumbar adj degenerative adj disease) .ti,ab.

12. (low adj back adj pain) .ti,ab.

13. (disc adj degeneration).ti,ab.

14. (disc adj prolapse).ti,ab.

15. (disc adj herniation).ti,ab.

16. (facet adj joints).ti,ab.

17. (non-fusion or nonfusion).ti,ab.

18. fusion.ti,ab.

19. (non-heal$ or nonheal$).ti,ab.

20. (heal or healed or heals or healing).ti,ab.

21. (allograft$ or autograft$ or autogenous or iliac crest).ti,ab.

22. (or/1-6) and (or/7-21)

23. randomized controlled trial.pt.

24. controlled clinical trial.pt.

25. comparative study.pt.

26. clinical trial.pt.

27. randomized.ab.

28. placebo.ab,ti.

29. drug therapy.fs.

30. randomly.ab,ti.

31. trial.ab,ti.

32. groups.ab,ti.

33. or/23-32

34. (animals not (humans and animals)).sh.

35. 33 not 34

36. and/22,35

*Embase search strategy*

1 ((bone morphogen$ or osteogen$ or osteoinduct$) adj (protein$ or factor$ or polypeptide$ or poly-peptide$)).ti,ab.

2 (BMP2 or BMP-2 or rhBMP2 or rhBMP-2 or rh-BMP2 or rh-BMP-2).ti,ab.

3 (BMP7 or BMP-7 or rhBMP7 or rhBMP-7 or rh-BMP7 or rh-BMP-7).ti,ab.

4 (OP1 or OP-1 or rhOP1 or rhOP-1 or rh-OP1 or rh-OP-1).ti,ab.

5 (InductOs or InFuse).ti,ab.

6 Bone Morphogenetic Protein/ or Bone Morphogenetic Protein 2/ or Bone Morphogenetic Protein 7/

7 Osteogenic Protein/ or Osteogenic Protein 1/

8 exp spine disease/

9 exp spine fusion/

10 exp spine/

11 exp low back pain/

12 exp backache/

13 (spine or spinal or thoracic or lumbar or cervical).ti,ab.

14 (lumbar adj degenerative adj disease).mp

15 (disc adj degeneration).mp.

16 (disc adj prolapse).mp.

17 (disc adj herniation).mp.

18 (facet adj joints).mp.

19 (non-fusion or nonfusion).ti,ab.

20 fusion.mp.

21 (non-heal$ or nonheal$).ti,ab.

22 (heal or healed or heals or healing).ti,ab.

23 Healing Impairment/ or Bone Allograft/ or Autograft/

24 (allograft$ or autograft$ or autogenous or iliac crest).ti,ab.

25 (or/1-7) and (or/8-24)

26 Clinical Article/

27 exp Clinical Study/

28 Clinical Trial/

29 Controlled Study/

30 Randomized Controlled Trial/

31 Major Clinical Study/

32 Double Blind Procedure/

33 Multicenter Study/

34 Single Blind Procedure/

35 Phase 3 Clinical Trial/

36 Phase 4 Clinical Trial/

37 crossover procedure/

38 placebo/

39 or/26-38

40 allocat$.mp.

41 assign$.mp.

42 blind$.mp.

43 (clinic$ adj25 (study or trial)).mp.

44 compar$.mp.

45 control$.mp.

46 cross?over.mp.

47 factorial$.mp.

48 follow?up.mp.

49 placebo$.mp.

50 prospectiv$.mp.

51 random$.mp.

52 ((singl$ or doubl$ or trebl$ or tripl$) adj25 (blind$ or mask$)).mp.

53 trial.mp.

54 (versus or vs).mp.

55 or/40-54

56 39 and 55

57 exp animals/ or exp invertebrate/ or animal experiment/ or animal model/ or animal tissue/ or animal cell/ or nonhuman/

58 human/ or normal human/ or human cell/

59 57 and 58

60 57 not 59

61 56 not 60

62 and/25, 61

*CENTRAL search strategy*

#1 MeSH descriptor Back Pain explode all trees

#2 dorsalgia

#3 backache

#4 MeSH descriptor Low Back Pain explode all trees

#5 (lumbar next pain) or (sciatica) or (spondylosis)

#6 MeSH descriptor Spine explode all trees

#7 MeSH descriptor Spinal Diseases explode all trees

#8 (lumbago) or (disc near degeneration) or (disc near prolapse) or (disc near herniation)

#9 spinal fusion

#10 back near pain

#11 facet near joints

#12 MeSH descriptor Intervertebral Disk explode all trees

#13 postlaminectomy

#14 sciatic*

#15 spinal disorder*

#16 MeSH descriptor Cauda Equina explode all trees

#17 lumbar near vertebra*

#18 spinal near stenosis

#19 slipped near (disc* or disk*)

#20 degenerat* near (disc* or disk*)

#21 stenosis near (spine or root or spinal)

#22 displace* near (disc* or disk*)

#23 prolap* near (disc* or disk*)

#24 MeSH descriptor Sciatic Neuropathy explode all trees

#25 (#1 OR #2 OR #3 OR #4 OR #5 OR #6 OR #7 OR #8 OR #9 OR #10 OR #11 OR #12 OR #13 OR #14 OR #15 OR #16 OR #17 OR #18 OR #19 OR #20 OR #21 OR #22 OR #23 OR #24)

#26 MeSH descriptor Bone Morphogenetic Proteins explode all trees

#27 (bone next morphogen* next protein*):ti,ab

#28 (bone next morphogen* next factor*):ti,ab

#29 (bone next morphogen* next polypeptide*):ti,ab

#30 (bone next morphogen* next poly-peptide*):ti,ab

#31 (osteogen* next protein*):ti,ab

#32 (osteogen* next factor*):ti,ab

#33 (osteogen* next polypeptide*):ti,ab

#34 (osteogen* next poly-peptide*):ti,ab

#35 (osteoinduct* next protein*):ti,ab

#36 (osteoinduct* next factor*):ti,ab

#37 (osteoinduct* next polypeptide*):ti,ab

#38 (osteoinduct* next poly-peptide*):ti,ab

#39(BMP2 or BMP-2 or rhBMP2 or rhBMP-2 or rh-BMP2 or rh-BMP-2).ti,ab.

#40 (BMP7 or BMP-7 or rhBMP7 or rhBMP-7 or rh-BMP7 or rh-BMP-7).ti,ab.

#41(OP1 or OP-1 or rhOP1 or rhOP-1 or rh-OP1 or rh-OP-1).ti,ab.

#42 (InductOs or InFuse).ti,ab.

#43 (#26 OR #27 OR #28 OR #29 OR #30 OR #31 OR #32 OR #33 OR #34 OR #35 OR #36 OR #37 OR #38 OR #39 OR #40 OR #41 OR #42)

#44 (#43 AND #25)
