# Supplementary material for: A Meta Analysis of Lumbar Spinal Fusion Surgery Using Bone Morphogenetic Proteins and Autologous Iliac Crest Bone Graft
Source: PLoS One. 2014 Jun 2;9(6):e97049. doi: 10.1371/journal.pone.0097049 (PMC4041715; doi:10.1371/journal.pone.0097049)
Supplement: List S1 — List of included-excluded studies. (DOC) [file pone.0097049.s003.doc]

List of included studies (sort by year of study)

| Boden SD, Zdeblick TA, Sandhu HS, et al. (2000) The use of rhBMP-2 in interbody fusion cages. Definitive evidence of osteoinduction in humans: a preliminary report. Spine 25:376-381. doi:10.1097/00007632-200002010-00020. |
| --- |
| Boden SD, Kang J, Sandhu H, et al. (2002) Use of recom-binant human bone morphogenetic protein-2 to achieve posterolateral lumbar spine fusion in humans: a prospective, randomized clinical pilot trial: 2002 Volvo Award in clinical studies. Spine 27:2662–2673. doi:10.1097/00007632- 200212010-00005. |
| Burkus JK, Gornet MF, Dickman CA, et al. (2002) Anterior lumbar interbody fusion using rhBMP-2 with tapered interbody cages. J Spinal Disord Tech 15:337–349. doi:10.1097/00024720-200210000- 00001. |
| Burkus JK, Transfeldt EE, Kitchel SH, et al. (2002) Clinical and radiographic outcomes of anterior lumbar interbody fusion using recombinant human bone morphogenetic protein-2. Spine 27:2396–2408. doi:10.1097/00007632-200211010-00015. |
| [Johnsson R](http://www.ncbi.nlm.nih.gov/pubmed?term=Johnsson R[Author]&cauthor=true&cauthor_uid=12461391), [Strömqvist B](http://www.ncbi.nlm.nih.gov/pubmed?term=Str?mqvist B[Author]&cauthor=true&cauthor_uid=12461391), [Aspenberg P](http://www.ncbi.nlm.nih.gov/pubmed?term=Aspenberg P[Author]&cauthor=true&cauthor_uid=12461391) (2002) Randomized radiostereometric study comparing osteogenic protein-1 (BMP-7) and autograft bone in human noninstrumented posterolateral lumbar fusion: 2002 Volvo Award in clinical studies. spine 27:2654-2661. doi:[10.1097/00007632- 200212010-00004](http://dx.doi.org/10.1097/00007632-200212010-00004). |
| Burkus JK, Dorchak JD, Sanders DL (2003) Radiographic assessment of interbody fusion using recombinant human bone morphogenetic protein type 2. Spine 28:372–377. doi:10.1097/01.BRS. 0000048469.45035.B9. |
| Haid RW Jr, Branch CL Jr, Alexander JT, et al. (2004) Posterior lumbar interbody fusion using recombinant human bone morphogenetic protein type 2 with cylindrical interbody cages. Spine J 4:527–538. doi:10.1016/j.spinee.2004.03.025 |
| Assiri I, du Plessis S, Hurlbert J, et al. (2004) A prospective randomized clinical study comparing instrumented lumbar fusion rates of Recombinant Human Bone Morphogenic Protein-2 (rhBMP-2) with autogenous iliac crest bone graft in patients with symptomatic degenerative disc disease. Canadian Journal of Surgery 47(Suppl 4):7-8. |
| Glassman SD, Dimar JR, Carreon LY, et al. (2005) Initial fusion rates with recombinant human bone morphogenetic protein-2/compression resistant matrix and a hydroxyapatite and tricalcium phosphate/collagen carrier in posterolateral spinal fusion. Spine 30:1694–1698. doi:10.1097/01. brs.0000172157.39513.80. |
| Vaccaro AR, Anderson DG, Patel T, et al. (2005) Comparison of OP-1 Putty (rhBMP-7) to iliac crest autograft for posterolateral lumbar arthrodesis: a minimum 2-year follow-up pilot study. Spine 30:2709-2716. [doi:10.1097/01.brs.0000190812.08447.ba](http://dx.doi.org/10.1097/01.brs.0000190812.08447.ba). c |
| Burkus JK, Sandhu HS, Gornet MF, et al. (2005) Use of rhBMP-2 in combination with structural cortical allografts: clinical and radiographic outcomes in anterior lumbar spinal surgery. J Bone Joint Surg Am 87:1205–1212. doi:10.2106/JBJS.D.02532. c |
| Dimar JR, Glassman SD, Burkus KJ, et al. (2006) Clinical outcomes and fusion success at 2 years of single-level instrumented posterolateral fusions with recombinant human bone morphogenetic protein-2/compression resistant matrix versus iliac crest bone graft. Spine 31:2534–2539. doi:10.1097/01.brs.0000240715.78657.81. |
| Kanayama M, Hashimoto T, Shigenobu K, et al. (2006) A prospective randomized study of posterolateral lumbar fusion using osteogenic protein-1 (OP-1) versus local autograft with ceramic bone substitute: emphasis of surgical exploration and histologic assessment. Spine 31:1067-1074. doi:10.1097/01.brs.0000216444.01888.21. |
| Glassman SD, Carreon LY, Djurasovic M, et al. (2008) RhBMP-2 versus iliac crest bone graft for lumbar spine fusion: a randomized, controlled trial in patients over 60 years of age. Spine 33:2843–2849. doi:10.1097/BRS.0b013e318190705d. |
| Vaccaro AR, Lawrence JP, Patel T, et al. (2008) The safety and efficacy of OP-1 (rhBMP-7) as a replacement for iliac crest autograft in posterolateral lumbar arthrodesis: a long-term (&gt;4 years) pivotal study. Spine 33:2850-2862. doi: 10.1097/BRS.0b013e31818a314d. |
| Dimar JR 2nd, Glassman SD, Burkus JK, et al. (2009) Clinical and radiographic analysis of an optimized rhBMP-2 formulation as an autograft replacement in posterolateral lumbar spine arthrodesis. Journal Bone Joint Surg Am 91:1377–1386. doi:10.2106/JBJS.H.00200. |
| Dawson E, Bae HW, Burkus JK, et al. (2009) Recombinant human bone morphogenetic protein-2 on an absorbable collagen sponge with an osteoconductive bulking agent in posterolateral arthrodesis with instrumentation. A prospective randomized trial. J Bone Joint Surg Am 91:1604–1613. doi:10.2106/ JBJS.G.01157. |
| Delawi D, Dhert WJ, Rillardon L, et al. (2010) A prospective, randomized, controlled, multicenter study of osteogenic protein-1 in instrumented posterolateral fusions: report on safety and feasibility. Spine 35:1185-1191. doi: 10.1097/BRS.0b013e3181d3cf28. |
| Michielsen J, Sys J, Rigaux A, et al. (2013) [The effect of recombinant human bone morphogenetic protein-2 in single-level posteriorlumbar interbody arthrodesis.](http://www.ncbi.nlm.nih.gov/pubmed/23677353) J Bone Joint Surg Am 95:873-880. doi: 10.2106/JBJS.L.00137. |

List of excluded studies (alphabetical order)

| An HS, Simpson JM, Glover JM, et al. Comparison between allograft plus demineralized bone matrix versus autograft in anterior cervical fusion. A prospective multicenter study. Spine (Phila Pa 1976). 1995 Oct 15;20(20):2211-6.  **Reason for exclusion: not about lumbar spine** |
| --- |
| Abdullah KG, Steinmetz MP, Benzel EC,et al. The state of lumbar fusion extenders. Spine (Phila Pa 1976). 2011 Sep 15;36(20):E1328-34.  **Reason for exclusion:review article** |
| Agarwal R, Williams K, Umscheid CA,et al. Osteoinductive bone graft substitutes for lumbar fusion: a systematic review. J Neurosurg Spine. 2009 Dec;11(6):729-40.  **Reason for exclusion:review article** |
| Ahlquist DA, Zou H, Domanico M,et al. Next-generation stool DNA test accurately detects colorectal cancer and large adenomas. Gastroenterology. 2012 Feb;142(2):248-56; quiz e25-6.  **Reason for exclusion:not about spine** |
| Alonso N, Tanikawa DY, Freitas Rda S,et al. Evaluation of maxillary alveolar reconstruction using a resorbable collagen sponge with recombinant human bone morphogenetic protein-2 in cleft lip and palate patients. Tissue Eng Part C Methods. 2010 Oct;16(5):1183-9.  **Reason for exclusion: not about spine** |
| Alsaleh KA, Tougas CA, Roffey DM, et al. Osteoconductive bone graft extenders in posterolateral thoracolumbar spinal fusion: a systematic review. Spine (Phila Pa 1976). 2012 Jul 15;37(16):E993-1000.  **Reason for exclusion:review article** |
| Alt V, Donell ST, Chhabra A,et al. A health economic analysis of the use of rhBMP-2 in Gustilo-Anderson grade III open tibial fractures for the UK, Germany, and France. Injury. 2009 Dec;40(12):1269-75.  **Reason for exclusion: not about spine** |
| Anand N, Hamilton JF, Perri B, et al. Cantilever TLIF with structural allograft and RhBMP2 for correction and maintenance of segmental sagittal lordosis: long-term clinical, radiographic, and functional outcome. Spine (Phila Pa 1976). 2006 Sep 15;31(20):E748-53.  **Reason for exclusion:non-RCT** |
| Anastasilakis AD, Polyzos SA, Gkiomisi A,et al. Comparative effect of zoledronic acid versus denosumab on serum sclerostin and dickkopf-1 levels of naive postmenopausal women with low bone mass: a randomized, head-to-head clinical trial. J Clin Endocrinol Metab. 2013 Aug;98(8):3206-12.  **Reason for exclusion:not about spine** |
| Ardawi MS, Rouzi AA, Al-Sibiani SA,et al. High serum sclerostin predicts the occurrence of osteoporotic fractures in postmenopausal women: the Center of Excellence for Osteoporosis Research Study. J Bone Miner Res. 2012 Dec;27(12):2592-602.  **Reason for exclusion:not about BMPs** |
| Ardawi MS, Rouzi AA, Qari MH. Physical activity in relation to serum sclerostin, insulin-like growth factor-1, and bone turnover markers in healthy premenopausal women: a cross-sectional and a longitudinal study. J Clin Endocrinol Metab. 2012 Oct;97(10):3691-9.  **Reason for exclusion:not about spine** |
| Arner JW, Daffner SD. Bone graft extenders and substitutes in the thoracolumbar spine. Am J Orthop (Belle Mead NJ). 2012 May;41(5):230-5.  **Reason for exclusion:review article** |
| Aro HT, Govender S, Patel AD, et al. Recombinant human bone morphogenetic protein-2: a randomized trial in open tibial fractures treated with reamed nail fixation. J Bone Joint Surg Am. 2011 May 4;93(9):801-8.  **Reason for exclusion:not about spine** |
| Arrabal PM, Visser R, Santos-Ruiz L,et al. Osteogenic molecules for clinical applications: improving the BMP-collagen system. Biol Res. 2013;46(4):421-9.  **Reason for exclusion:non-RCT** |
| Asano N, Yamakazi T, Seto M, The expression and prognostic significance of bone morphogenetic protein-2 in patients with malignant fibrous histiocytoma. J Bone Joint Surg Br. 2004 May;86(4):607-12.  **Reason for exclusion:not about spine** |
| Ashley-Koch AE, Elliott L, Kail ME, et al. Identification of genetic polymorphisms associated with risk for pulmonary hypertension in sickle cell disease. Blood. 2008 Jun 15;111(12):5721-6.  **Reason for exclusion: not about spine** |
| Axelrad TW, Einhorn TA. Bone morphogenetic proteins in orthopaedic surgery.  Cytokine Growth Factor Rev. 2009 Oct-Dec;20(5-6):481-8.  **Reason for exclusion:review article** |
| Axelsson P, Johnsson R, Strömqvist B. Radiostereometry in lumbar spine research. Acta Orthop Suppl. 2006 Oct;77(323):1-42.  **Reason for exclusion:non-RCT** |
| Baskin DS, Ryan P, Sonntag V, et al. A prospective, randomized, controlled cervical fusion study using recombinant human bone morphogenetic protein-2 with the CORNERSTONE-SR allograft ring and the ATLANTIS anterior cervical plate. Spine (Phila Pa 1976). 2003 Jun 15;28(12):1219-24  **Reason for exclusion: not about lumbar spine** |
| Becker W, Clokie C, Sennerby L, Histologic findings after implantation and evaluation of different grafting materials and titanium micro screws into extraction sockets: case reports. J Periodontol. 1998 Apr;69(4):414-21.  **Reason for exclusion: non-RCT** |
| Benglis D, Wang MY, Levi AD. A comprehensive review of the safety profile of bone morphogenetic protein in spine surgery. Neurosurgery. 2008 May;62(5 Suppl 2):ONS423-31; discussion ONS431.  **Reason for exclusion:review article** |
| Bergström I, Parini P, Gustafsson SA, et al. Physical training increases osteoprotegerin in postmenopausal women. J Bone Miner Metab. 2012 Mar;30(2):202-7.  **Reason for exclusion:not about spine** |
| Bilic R, Simic P, Jelic M,et al. Osteogenic protein-1 (BMP-7) accelerates healing of scaphoid non-union with proximal pole sclerosis. Int Orthop. 2006 Apr;30(2):128-34. Epub 2006 Feb 28.  **Reason for exclusion: not about spine** |
| Bianchi J, Fiorellini JP, Howell TH,et al. Measuring the efficacy of rhBMP-2 to regenerate bone: a radiographic study using a commercially available software program. Int J Periodontics Restorative Dent. 2004 Dec;24(6):579-87.  **Reason for exclusion:non-RCT** |
| Boakye M, Mummaneni PV, Garrett M,et al. Anterior cervical discectomy and fusion involving a polyetheretherketone spacer and bone morphogenetic protein. J Neurosurg Spine. 2005 May;2(5):521-5.  **Reason for exclusion:not about lumbar spine** |
| Boden SD, Grob D, Damien C. Ne-Osteo bone growth factor for posterolateral lumbar spine fusion: results from a nonhuman primate study and a prospective human clinical pilot study. Spine (Phila Pa 1976). 2004 Mar 1;29(5):504-14.  **Reason for exclusion: non-human study** |
| Bond AM, Bhalala OG, Kessler JA. The dynamic role of bone morphogenetic proteins in neural stem cell fate and maturation. Dev Neurobiol. 2012 Jul;72(7):1068-84.  **Reason for exclusion:review article** |
| Bong MR, Capla EL, Egol KA,et al. Osteogenic protein-1 (bone morphogenic protein-7) combined with various adjuncts in the treatment of humeral diaphyseal nonunions. Bull Hosp Jt Dis. 2005;63(1-2):20-3.  **Reason for exclusion: not about spine** |
| Boyne PJ, Lilly LC, Marx RE,et al. De novo bone induction by recombinant human bone morphogenetic protein-2 (rhBMP-2) in maxillary sinus floor augmentation. J Oral Maxillofac Surg. 2005 Dec;63(12):1693-707.  **Reason for exclusion: not about spine** |
| Boyne PJ, Marx RE, Nevins M, et al. A feasibility study evaluating rhBMP-2/absorbable collagen sponge for maxillary sinus floor augmentation. Int J Periodontics Restorative Dent. 1997 Feb;17(1):11-25.  **Reason for exclusion:not about spine** |
| Brandoff JF, Silber JS, Vaccaro AR. Contemporary alternatives to synthetic bone grafts for spine surgery. Am J Orthop (Belle Mead NJ). 2008 Aug;37(8):410-4.  **Reason for exclusion:review article** |
| Brannian J, Eyster K, Mueller BA,et al. Differential gene expression in human granulosa cells from recombinant FSH versus human menopausal gonadotropin ovarian stimulation protocols. Reprod Biol Endocrinol. 2010 Mar 12;8:25.  **Reason for exclusion: not about spine** |
| Brownfield LA, Weltman RL. Ridge preservation with or without an osteoinductive allograft: a clinical, radiographic, micro-computed tomography, and histologic study evaluating dimensional changes and new bone formation of the alveolar ridge. J Periodontol. 2012 May;83(5):581-9.  **Reason for exclusion: not about spine** |
| Brounts SH, Lee JS, Weinberg S, et al. High affinity binding of an engineered, modular peptide to bone tissue. Mol Pharm. 2013 May 6;10(5):2086-90.  **Reason for exclusion:non-RCT** |
| Burkus JK. Bone morphogenetic proteins in anterior lumbar interbody fusion: old techniques and new technologies. Invited submission from the Joint Section Meeting on Disorders of the Spine and Peripheral Nerves, March 2004.J Neurosurg Spine. 2004 1:254-260.  **Reason for exclusion: duplicate report** |
| Burkus JK, Dryer RF, Peloza JH. Retrograde ejaculation following single-level anterior lumbar surgery with or without recombinant human bone morphogenetic protein-2 in 5 randomized controlled trials: clinical article. J Neurosurg Spine. 2013 Feb;18(2):112-21.  **Reason for exclusion:non-RCT** |
| Burkus JK, Gornet MF, Glassman SD, et al. Blood serum antibody analysis and long-term follow-up of patients treated with recombinant human bone morphogenetic protein-2 in the lumbar spine.Spine 2011 36:2158-2167.  **Reason for exclusion:non-RCT** |
| Burkus JK, Gornet MF, Schuler TC, Six-year outcomes of anterior lumbar interbody arthrodesis with use of interbodyfusion cages and recombinant human bone morphogenetic protein-2.J Bone Joint Surg Am 2009 91:1181-1189.  **Reason for exclusion:serial report in different follows** |
| Burkus JK, Sandhu HS, Gornet MF.Influence of rhBMP-2 on the healing patterns associated with allograft interbody constructs in comparison with autograft.Spine 2006 31:775-781.  **Reason for exclusion:serial report in different follows** |
| Buser D, Dula K, Hirt HP, et al. Lateral ridge augmentation using autografts and barrier membranes: a clinical study with 40 partially edentulous patients. J Oral Maxillofac Surg. 1996 Apr;54(4):420-32; discussion 432-3.  **Reason for exclusion: not about spine** |
| Buttermann GR. Prospective nonrandomized comparison of an allograft with bone morphogenic protein versus an iliac-crest autograft in anterior cervical discectomy and fusion. Spine J. 2008 May-Jun;8(3):426-35. Epub 2007 Mar 7.  **Reason for exclusion:non-RCT** |
| Cabraja M, Kroppenstedt S. Bone grafting and substitutes in spine surgery. J Neurosurg Sci. 2012 Jun;56(2):87-95.  **Reason for exclusion:review article** |
| Calori GM, D'Avino M, Tagliabue L,et al. An ongoing research for evaluation of treatment with BMPs or AGFs in long bone non-union: protocol description and preliminary results. Injury. 2006 Sep;37 Suppl 3:S43-50.  **Reason for exclusion: not about spine** |
| Calori GM, Tagliabue L, Gala L,et al. Application of rhBMP-7 and platelet-rich plasma in the treatment of long bone non-unions: a prospective randomised clinical study on 120 patients. Injury. 2008 Dec;39(12):1391-402.  **Reason for exclusion: not about spine** |
| Canan LW Jr, da Silva Freitas R, Alonso N, et al. Human bone morphogenetic protein-2 use for maxillary reconstruction in cleft lip and palate patients. J Craniofac Surg. 2012 Nov;23(6):1627-33.  **Reason for exclusion:not about spine** |
| Camargo PM, Wolinsky LE, Burgess AV, et al.Bovine-derived bone protein extract in the treatment of mandibular Class II furcations. Compend Contin Educ Dent. 2002 Nov;23(11):1023-8, 1030, 1032 passim; quiz 1042.  **Reason for exclusion:not about spine** |
| Carreon LY, Glassman SD, Djurasovic M, et al.RhBMP-2 versus iliac crest bone graft for lumbar spine fusion in patients over 60 years of age: a cost-utility study.Spine 2009 34:238-243.  **Reason for exclusion:serial report in different follows** |
| Carragee EJ, Bono CM, Scuderi GJ. Pseudomorbidity in iliac crest bone graft harvesting: the rise of rhBMP-2 in short-segment posterior lumbar fusion. Spine J. 2009 Nov;9(11):873-9.  **Reason for exclusion:review article** |
| Carragee EJ, Chu G, Rohatgi R,et al. Cancer risk after use of recombinant bone morphogenetic protein-2 for spinal arthrodesis. J Bone Joint Surg Am. 2013 Sep 4;95(17):1537-45.  **Reason for exclusion: different comparison** |
| Carragee EJ, Hurwitz EL, Weiner BK. A critical review of recombinant human bone morphogenetic protein-2 trials in spinal surgery: emerging safety concerns and lessons learned. Spine J. 2011 Jun;11(6):471-91.  **Reason for exclusion:review article** |
| Catalano A, Morabito N, Basile G, et al. Zoledronic acid acutely increases sclerostin serum levels in women with postmenopausal osteoporosis. J Clin Endocrinol Metab. 2013 May;98(5):1911-5.  **Reason for exclusion:not about spine** |
| Chand AL, Ponnampalam AP, Harris SE,et al. Mutational analysis of BMP15 and GDF9 as candidate genes for premature ovarian failure. Fertil Steril. 2006 Oct;86(4):1009-12.  **Reason for exclusion: not about spine** |
| Chen JR, Lazarenko OP, Blackburn ML, et al. Infant formula promotes bone growth in neonatal piglets by enhancing osteoblastogenesis through bone morphogenic protein signaling. J Nutr. 2009 Oct;139(10):1839-47.  **Reason for exclusion: vitro experiment** |
| Cheng I, Krumroy LM, Plummer SJ, et al. MIC1 and IL1RN genetic variation and advanced prostate cancer risk. Cancer Epidemiol Biomarkers Prev. 2007 Jun;16(6):1309-11.  **Reason for exclusion: not about spine** |
| Cho SK, Stoker GE, Bridwell KH. Spinal reconstruction with pedicle screw-based instrumentation and rhBMP-2 in patients with neurofibromatosis and severe dural ectasia and spinal deformity: report of two cases and a review of the literature. J Bone Joint Surg Am. 2011 Aug 3;93(15):e86.  **Reason for exclusion:review article** |
| Chrastil J, Low JB, Whang PG,et al. Complications associated with the use of the recombinant human bone morphogenetic proteins for posterior interbody fusions of the lumbar spine. Spine (Phila Pa 1976). 2013 Jul 15;38(16):E1020-7.  **Reason for exclusion:review article** |
| Chrastil J, Patel AA. Complications associated with posterior and transforaminal lumbar interbody fusion. J Am Acad Orthop Surg. 2012 May;20(5):283-91.  **Reason for exclusion:review article** |
| Chuengsamarn S, Rattanamongkoulgul S, Suwanwalaikorn S,et al. Effects of statins vs. non-statin lipid-lowering therapy on bone formation and bone mineral density biomarkers in patients with hyperlipidemia. Bone. 2010 Apr;46(4):1011-5.  **Reason for exclusion: not about spine** |
| Cidem M, Usta TA, Karacan I,et al. Effects of sex steroids on serum sclerostin levels during the menstrual cycle. Gynecol Obstet Invest. 2013;75(3):179-84.  **Reason for exclusion:not about spine** |
| Cipitria A, Reichert JC, Epari DR,et al. Polycaprolactone scaffold and reduced rhBMP-7 dose for the regeneration of critical-sized defects in sheep tibiae. Biomaterials. 2013 Dec;34(38):9960-8.  **Reason for exclusion:animal model** |
| Ciumas M, Eyries M, Poirier O,et al. Bone morphogenetic proteins protect pulmonary microvascular endothelial cells from apoptosis by upregulating α-B-crystallin. Arterioscler Thromb Vasc Biol. 2013 Nov;33(11):2577-84.  **Reason for exclusion:not about spine** |
| Cochran DL, Jones AA, Lilly LC, Evaluation of recombinant human bone morphogenetic protein-2 in oral applications including the use of endosseous implants: 3-year results of a pilot study in humans. J Periodontol. 2000 Aug;71(8):1241-57.  **Reason for exclusion:not about spine** |
| Corinaldesi G, Piersanti L, Piattelli A,et al. Augmentation of the floor of the maxillary sinus with recombinant human bone morphogenetic protein-7: a pilot radiological and histological study in humans. Br J Oral Maxillofac Surg. 2013 Apr;51(3):247-52.  **Reason for exclusion:not about spine** |
| Coseo NM, Saldua N, Harrop J. Current use of biologic graft extenders for spinal fusion. J Neurosurg Sci. 2012 Sep;56(3):203-7.  **Reason for exclusion:review article** |
| Culpepper BK, Webb WM, Bonvallet PP, et al. Tunable delivery of bioactive peptides from hydroxyapatite biomaterials and allograft bone using variable-length polyglutamate domains. J Biomed Mater Res A. 2013 Apr 27.  **Reason for exclusion: not about spine** |
| David SM, Gruber HE, Meyer RA Jr,et al. Lumbar spinal fusion using recombinant human bone morphogenetic protein in the canine. A comparison of three dosages and two carriers. Spine (Phila Pa 1976). 1999 Oct 1;24(19):1973-9.  **Reason for exclusion:animal model** |
| D'Agostino P, Barbier O. An investigation of the effect of AlloMatrix bone graft in distal radial fracture: a prospective randomised controlled clinical trial. Bone Joint J. 2013 Nov;95-B(11):1514-20.  **Reason for exclusion: not about spine** |
| Demmers KJ, Smaill B, Davis GH, et al. Heterozygous Inverdale ewes show increased ovulation rate sensitivity to pre-mating nutrition. Reprod Fertil Dev. 2011;23(7):866-75.  **Reason for exclusion:non-human study** |
| de Oliveira EM, Martinez EF, Bossonaro JP, et al. In-vitro analysis of rhBMP-2 effects in human osteogenic cells. Can J Physiol Pharmacol. 2013 Nov;91(11):929-34.  **Reason for exclusion: not about spine** |
| Devaney JM, Tosi LL, Fritz DT,et al. Differences in fat and muscle mass associated with a functional human polymorphism in a post-transcriptional BMP2 gene regulatory element. J Cell Biochem. 2009 Aug 15;107(6):1073-82.  **Reason for exclusion:non-RCT** |
| Dickinson BP, Ashley RK, Wasson KL, et al. Reduced morbidity and improved healing with bone morphogenic protein-2 in older patients with alveolar cleft defects. Plast Reconstr Surg. 2008 Jan;121(1):209-17.  **Reason for exclusion: not about spine** |
| Dmitriev AE, Lehman RA Jr, Symes AJ. Bone morphogenetic protein-2 and spinal arthrodesis: the basic science perspective on protein interaction with the nervous system. Spine J. 2011 Jun;11(6):500-5.  **Reason for exclusion:review article** |
| Doak SH, Jenkins SA, Hurle RA,et al. Bone morphogenic factor gene dosage abnormalities in prostatic intraepithelial neoplasia and prostate cancer. Cancer Genet Cytogenet. 2007 Jul 15;176(2):161-5.  **Reason for exclusion: not about spine** |
| Dooley C A, Attia G R, Rainey W E. Bone morphogenetic protein inhibits ovarian androgen production. Journal of Clinical Endocrinology and Metabolism,2000,85(09):3331-3337. DOI:10.1210/jc.85.9.3331.  **Reason for exclusion:not about spine** |
| Drake MT, Srinivasan B, Mödder UI,et al. Effects of parathyroid hormone treatment on circulating sclerostin levels in postmenopausal women. J Clin Endocrinol Metab. 2010 Nov;95(11):5056-62.  **Reason for exclusion: not about spine** |
| Dunworth WP, Cardona-Costa J, Bozkulak EC, Bone morphogenetic protein 2 signaling negatively modulates lymphatic development in vertebrate embryos. Circ Res. 2014 Jan 3;114(1):56-66. doi: 10.1161/CIRCRESAHA.114.302452. Epub 2013 Oct 11.  **Reason for exclusion:non-RCT** |
| Ekrol I, Hajducka C, Court-Brown C,et al. A comparison of RhBMP-7 (OP-1) and autogenous graft for metaphyseal defects after osteotomy of the distal radius. Injury. 2008 Sep;39 Suppl 2:S73-82.  **Reason for exclusion: not about spine** |
| Even J, Eskander M, Kang J. Bone morphogenetic protein in spine surgery: current and future uses. J Am Acad Orthop Surg. 2012 Sep;20(9):547-52.  **Reason for exclusion:review article** |
| Fang Z, Sun T, Yadav SK. Research progress of bone morphogenetic protein and liability of ossification of posterior longitudinal ligament. Zhongguo Xiu Fu Chong Jian Wai Ke Za Zhi. 2012 Oct;26(10):1255-8.  **Reason for exclusion:review article** |
| Faje AT, Fazeli PK, Katzman DK, et al. Sclerostin levels and bone turnover markers in adolescents with anorexia nervosa and healthy adolescent girls. Bone. 2012 Sep;51(3):474-9.  **Reason for exclusion:not about spine** |
| Faria ML, Lu Y, Heaney K, et al. Recombinant human bone morphogenetic protein-2 in absorbable collagen sponge enhances bone healing of tibial osteotomies in dogs. Vet Surg. 2007 Feb;36(2):122-31.  **Reason for exclusion:animal model.** |
| Ferreira JR, Padilla R, Urkasemsin G,et al. Titanium-enriched hydroxyapatite-gelatin scaffolds with osteogenically differentiated progenitor cell aggregates for calvaria bone regeneration. Tissue Eng Part A. 2013 Aug;19(15-16):1803-16.  **Reason for exclusion: not about spine** |
| Ferretti C, Ripamonti U. Human segmental mandibular defects treated with naturally derived bone morphogenetic proteins. J Craniofac Surg. 2002 May;13(3):434-44.  **Reason for exclusion:not about spine** |
| Fernández-Rozadilla C, de Castro L, Clofent J, et al.Single nucleotide polymorphisms in the Wnt and BMP pathways and colorectal cancer risk in a Spanish cohort. PLoS One. 2010 Sep 9;5(9). pii: e12673. **Reason for exclusion: not about spine** |
| Fernandez-Rozadilla C, Palles C, Carvajal-Carmona L, et al.BMP2/BMP4 colorectal cancer susceptibility loci in northern and southern European populations. Carcinogenesis. 2013 Feb;34(2):314-8.  **Reason for exclusion:not about spine** |
| Fiorellini JP, Howell TH, Cochran D, Randomized study evaluating recombinant human bone morphogenetic protein-2 for extraction socket augmentation. J Periodontol. 2005 Apr;76(4):605-13.  **Reason for exclusion:not about spine** |
| Friedlaender GE, Perry CR, Cole JD, et al. Osteogenic protein-1 (bone morphogenetic protein-7) in the treatment of tibial nonunions. J Bone Joint Surg Am. 2001;83-A Suppl 1(Pt 2):S151-8.  **Reason for exclusion:not about spine** |
| Friedlaender GE. Osteogenic protein-1 in treatment of tibial nonunions: current status. Surg Technol Int. 2004;13:249-52.  **Reason for exclusion:not about spine** |
| Furlan JC, Perrin RG, Govender PV, et al. Use of osteogenic protein-1 in patients at high risk for spinal pseudarthrosis: a prospective cohort study assessing safety, health-related quality of life, and radiographic fusion. Invited submission from the Joint Section on Disorders of the Spine and Peripheral Nerves, March 2007. J Neurosurg Spine. 2007 Nov;7(5):486-95.  **Reason for exclusion:non-RCT** |
| Gatti D, Viapiana O, Idolazzi L,et al. The waning of teriparatide effect on bone formation markers in postmenopausal osteoporosis is associated with increasing serum levels of DKK1. J Clin Endocrinol Metab. 2011 May;96(5):1555-9.  **Reason for exclusion:not about spine** |
| Gatti D, Viapiana O, Adami S,et al. Bisphosphonate treatment of postmenopausal osteoporosis is associated with a dose dependent increase in serum sclerostin. Bone. 2012 Mar;50(3):739-42.  **Reason for exclusion:not about spine** |
| Gatti D, Viapiana O, Fracassi E,et al. Sclerostin and DKK1 in postmenopausal osteoporosis treated with denosumab. J Bone Miner Res. 2012 Nov;27(11):2259-63.  **Reason for exclusion:not about spine** |
| Geesink RG, Hoefnagels NH, Bulstra SK. Osteogenic activity of OP-1 bone morphogenetic protein (BMP-7) in a human fibular defect. J Bone Joint Surg Br. 1999 Jul;81(4):710-8.  **Reason for exclusion:not about spine** |
| Giannoudis PV, Kanakaris NK, Dimitriou R,et al. The synergistic effect of autograft and BMP-7 in the treatment of atrophic nonunions. Clin Orthop Relat Res. 2009 Dec;467(12):3239-48.  **Reason for exclusion: not about spine** |
| Glassman SD, Dimar JR 3rd, Burkus K, et al.The efficacy of rhBMP-2 for posterolateral lumbar fusion in smokers. Spine 2007 32:1693-1698.  **Reason for exclusion:****non-RCT** |
| Glassman SD, Carreon LY, Campbell MJ, et al.The perioperative cost of Infuse bone graft in posterolateral lumbar spine fusion.Spine J 2008 8:443-448.  **Reason for exclusion:serial report in different follows** |
| Glassman SD, Howard JM, Sweet A,et al. Complications and concerns with osteobiologics for spine fusion in clinical practice. Spine (Phila Pa 1976). 2010 Aug 1;35(17):1621-8.  **Reason for exclusion:review article** |
| Gónzalez A, Ramírez-Lorca R, Calatayud C, et al. Association of genetic markers within the BMP15 gene with anovulation and infertility in women with polycystic ovary syndrome. Fertil Steril. 2008 Aug;90(2):447-9. Epub 2007 Oct 1.  **Reason for exclusion: not about spine** |
| Grabowski G, Cornett CA. Bone graft and bone graft substitutes in spine surgery: current concepts and controversies. J Am Acad Orthop Surg. 2013 Jan;21(1):51-60.  **Reason for exclusion:review article** |
| Groeneveld EH, van den Bergh JP, Holzmann P, Histomorphometrical analysis of bone formed in human maxillary sinus floor elevations grafted with OP-1 device, demineralized bone matrix or autogenous bone. Comparison with non-grafted sites in a series of case reports. Clin Oral Implants Res. 1999 Dec;10(6):499-509.  **Reason for exclusion:not about spine** |
| Govender S, Csimma C, Genant HK, Recombinant human bone morphogenetic protein-2 for treatment of open tibial fractures: a prospective, controlled, randomized study of four hundred and fifty patients. J Bone Joint Surg Am. 2002 Dec;84-A(12):2123-34.  **Reason for exclusion:not about spine** |
| Guimarães Mdo C, Passanezi E, Sant'Ana AC, et al. Digital subtraction radiographic analysis of the combination of bioabsorbable membrane and bovine morphogenetic protein pool in human periodontal infrabony defects. J Appl Oral Sci. 2010 Jul-Aug;18(4):379-84.  **Reason for exclusion: not about spine** |
| Gupta R, Pandit N, Malik R, et al. Clinical and radiological evaluation of an osseous xenograft for the treatment of infrabony defects. J Can Dent Assoc. 2007 Jul-Aug;73(6):513.  **Reason for exclusion: not about spine** |
| Guskuma MH, Hochuli-Vieira E, Pereira FP,et al. Evaluation of the presence of VEGF, BMP2 and CBFA1 proteins in autogenous bone graft: Histometric and immunohistochemical analysis. J Craniomaxillofac Surg. 2013 Aug 7. pii: S1010-5182(13)00152-2.  **Reason for exclusion:non-RCT** |
| Hahn JY, Cho HJ, Kang HJ,et al. Pre-treatment of mesenchymal stem cells with a combination of growth factors enhances gap junction formation, cytoprotective effect on cardiomyocytes, and therapeutic efficacy for myocardial infarction. J Am Coll Cardiol. 2008 Mar 4;51(9):933-43.  **Reason for exclusion: not about spine** |
| Hayashi T, Kobayashi S, Asakura M, Immature muscular tissue differentiation into bone-like tissue by bone morphogenetic proteins in vitro, with ossification potential in vivo. J Biomed Mater Res A. 2013 Oct 1.  **Reason for exclusion: vitro experiment** |
| Howell TH, Fiorellini J, Jones A, et al. A feasibility study evaluating rhBMP-2/absorbable collagen sponge device for local alveolar ridge preservation or augmentation. Int J Periodontics Restorative Dent. 1997 Apr;17(2):124-39.  **Reason for exclusion:not about spine** |
| Howe JR, Sayed MG, Ahmed AF, et al. The prevalence of MADH4 and BMPR1A mutations in juvenile polyposis and absence of BMPR2, BMPR1B, and ACVR1 mutations. J Med Genet. 2004 Jul;41(7):484-91.  **Reason for exclusion:non-RCT** |
| Hodges SD, Eck JC, Newton D. Retrospective study of posterior cervical fusions with rhBMP-2. Orthopedics. 2012 Jun;35(6):e895-8.  **Reason for exclusion:not about lumbar spine** |
| Hwang CJ, Vaccaro AR, Hong J,et al. Immunogenicity of osteogenic protein 1: results from a prospective, randomized, controlled, multicenter pivotal study of uninstrumented lumbar posterolateral fusion. J Neurosurg Spine. 2010 Oct;13(4):484-93.  **Reason for exclusion: duplicate report** |
| Hunter DJ, Pike MC, Jonas BL, et al. Phase 1 safety and tolerability study of BMP-7 in symptomatic knee osteoarthritis. BMC Musculoskelet Disord. 2010 Oct 10;11:232.  **Reason for exclusion: not about spine** |
| Ishibashi O. Bone Morphogenetic Protein-2 Desensitizes MC3T3-E1 Osteoblastic Cells to Estrogen Through Transcriptional Downregulation of Estrogen Receptor 1. J Bone Metab. 2013 Nov;20(2):83-8  **Reason for exclusion: not about spine** |
| Jain A, Kebaish KM, Sponseller PD. Factors associated with use of bone morphogenetic protein during pediatric spinal fusion surgery: an analysis of 4817 patients. J Bone Joint Surg Am. 2013 Jul 17;95(14):1265-70.  **Reason for exclusion:non-RCT** |
| Jones AL. Recombinant human bone morphogenic protein-2 in fracture care. J Orthop Trauma. 2005 Nov-Dec;19(10 Suppl):S23-5.  **Reason for exclusion: not about spine** |
| Jones AL, Bucholz RW, Bosse MJ, et al. Recombinant human BMP-2 and allograft compared with autogenous bone graft for reconstruction of diaphyseal tibial fractures with cortical defects. A randomized, controlled trial. J Bone Joint Surg Am. 2006 Jul;88(7):1431-41.  **Reason for exclusion: not about spine** |
| Jo S, Kim S, Cho TH, et al. Effects of recombinant human bone morphogenic protein-2 and human bone marrow-derived stromal cells on in vivo bone regeneration of chitosan-poly(ethylene oxide) hydrogel. J Biomed Mater Res A. 2013 Mar;101(3):892-901.  **Reason for exclusion:non-RCT** |
| Jung RE1, Glauser R, Schärer P, Effect of rhBMP-2 on guided bone regeneration in humans. Clin Oral Implants Res. 2003 Oct;14(5):556-68.  **Reason for exclusion:non-RCT** |
| Jung RE, Windisch SI, Eggenschwiler AM, et al. A randomized-controlled clinical trial evaluating clinical and radiological outcomes after 3 and 5 years of dental implants placed in bone regenerated by means of GBR techniques with or without the addition of BMP-2. Clin Oral Implants Res. 2009 Jul;20(7):660-6.  **Reason for exclusion: not about spine** |
| Kanakaris NK, Calori GM, Verdonk R, et al. Application of BMP-7 to tibial non-unions: a 3-year multicenter experience. Injury. 2008 Sep;39 Suppl 2:S83-90.  **Reason for exclusion: not about spine** |
| Kanakaris NK, Lasanianos N, Calori GM, et al. Application of bone morphogenetic proteins to femoral non-unions: a 4-year multicentre experience. Injury. 2009 Dec;40 Suppl 3:S54-61.  **Reason for exclusion: not about spine** |
| Kanakaris NK, Giannoudis PV. Clinical applications of bone morphogenetic proteins: current evidence. J Surg Orthop Adv. 2008 Fall;17(3):133-46.  **Reason for exclusion:review article** |
| Kaji H, Imanishi Y, Sugimoto T,et al. Comparisons of serum sclerostin levels among patients with postmenopausal osteoporosis, primary hyperparathyroidism and osteomalacia. Exp Clin Endocrinol Diabetes. 2011 Jul;119(7):440-4.  **Reason for exclusion:not about spine** |
| Kao DW, Kubota A, Nevins M,et al. The negative effect of combining rhBMP-2 and Bio-Oss on bone formation for maxillary sinus augmentation. Int J Periodontics Restorative Dent. 2012 Feb;32(1):61-7.  **Reason for exclusion:not about spine** |
| Kariyawasam HH, Xanthou G, Barkans J, et al. Basal expression of bone morphogenetic protein receptor is reduced in mild asthma. Am J Respir Crit Care Med. 2008 May 15;177(10):1074-81.  **Reason for exclusion: not about spine** |
| Katayama Y, Matsuyama Y, Yoshihara H, et al. Clinical and radiographic outcomes of posterolateral lumbar spine fusion in humans using recombinant human bone morphogenetic protein-2: an average five-year follow-up study. Int Orthop. 2009 Aug;33(4):1061-7.  **Reason for exclusion:serial report in different follows** |
| Khurana JS, Ogino S, Shen T, Bone morphogenetic proteins are expressed by both bone-forming and non-bone-forming lesions. Arch Pathol Lab Med. 2004 Nov;128(11):1267-69.  **Reason for exclusion:not about spine** |
| Khurana S, Buckley S, Schouteden S, et al.A novel role of BMP4 in adult hematopoietic stem and progenitor cell homing via Smad independent regulation of integrin-α4 expression. Blood. 2013 Jan 31;121(5):781-90.  **Reason for exclusion:not about spine** |
| Kim M, Choe S. BMPs and their clinical potentials. BMB Rep. 2011 Oct;44(10):619-34.  **Reason for exclusion:review article** |
| Ko E, Yang K, Shin J, et al. Polydopamine-assisted osteoinductive peptide immobilization of polymer scaffolds for enhanced bone regeneration by human adipose-derived stem cells. Biomacromolecules. 2013 Sep 9;14(9):3202-13.  **Reason for exclusion: not about spine** |
| Kuklo TR, Rosner MK, Polly DW Jr. Computerized tomography evaluation of a resorbable implant after transforaminal lumbar interbody fusion. Neurosurg Focus. 2004 Mar 15;16(3):E10.  **Reason for exclusion:non-RCT** |
| Kurebayashi N, Sato M, Fujisawa T,et al. Regulation of neuropeptide Y Y1 receptor expression by bone morphogenetic protein 2 in C2C12 myoblasts. Biochem Biophys Res Commun. 2013 Oct 4;439(4):506-10.  **Reason for exclusion:non-RCT** |
| Lai RF, Zhou ZY, Chen T. Accelerating bone generation and bone mineralization in the Interparietal sutures of rats using an rhBMP-2/ACS composite after rapid expansion. Exp Anim. 2013;62(3):189-96.  **Reason for exclusion: animal model** |
| Lanman TH, Hopkins TJ. Lumbar interbody fusion after treatment with recombinant human bone morphogenetic protein-2 added to poly(L-lactide-co-D,L-lactide) bioresorbable implants. Neurosurg Focus. 2004 Mar 15;16(3):E9.  **Reason for exclusion:non-RCT** |
| Leach J, Bittar RG. BMP-7 (OP-1) safety in anterior cervical fusion surgery. J Clin Neurosci. 2009 Nov;16(11):1417-20.  **Reason for exclusion:non-RCT** |
| Le Dréau G, Martí E. The multiple activities of BMPs during spinal cord development. Cell Mol Life Sci. 2013 Nov;70(22):4293-305.  **Reason for exclusion:review article** |
| Lee FY, Sinicropi SM, Lee FS, et al. Treatment of congenital pseudarthrosis of the tibia with recombinant human bone morphogenetic protein-7 (rhBMP-7). A report of five cases. J Bone Joint Surg Am. 2006 Mar;88(3):627-33.  **Reason for exclusion: not about spine** |
| Liu B, Lun DX. Current application of β-tricalcium phosphate composites in orthopaedics. Orthop Surg. 2012 Aug;4(3):139-44.  **Reason for exclusion:review article** |
| Liu G, Kawaguchi H, Ogasawara T,et al. Optimal combination of soluble factors for tissue engineering of permanent cartilage from cultured human chondrocytes. J Biol Chem. 2007 Jul 13;282(28):20407-15. Epub 2007 May 10.  **Reason for exclusion: not about spine** |
| Lo KW, Ulery BD, Ashe KM,et al. Studies of bone morphogenetic protein-based surgical repair. Adv Drug Deliv Rev. 2012 Sep;64(12):1277-91.  **Reason for exclusion:review article** |
| Lyon T, Scheele W, Bhandari M,et al. Efficacy and safety of recombinant human bone morphogenetic protein-2/calcium phosphate matrix for closed tibial diaphyseal fracture: a double-blind, randomized, controlled phase-II/III trial. J Bone Joint Surg Am. 2013 Dec 4;95(23):2088-96.  **Reason for exclusion:not about spine** |
| Luhmann SJ, Bridwell KH, Cheng I,et al. Use of bone morphogenetic protein-2 for adult spinal deformity. Spine (Phila Pa 1976). 2005 Sep 1;30(17 Suppl):S110-7.  **Reason for exclusion:non-RCT** |
| Maniscalco P, Gambera D, Bertone C, Healing of fresh tibial fractures with OP-1. A preliminary report. Acta Biomed. 2002;73(1-2):27-33.  **Reason for exclusion:not about spine** |
| Maté-Sánchez de Val JE, Mazón P, Guirado JL, et al. Comparison of three hydroxyapatite/β-tricalcium phosphate/collagen ceramic scaffolds: An in vivo study. J Biomed Mater Res A. 2013 May 7.  **Reason for exclusion: not about spine** |
| Meisel HJ, Schnöring M, Hohaus C,et al. Posterior lumbar interbody fusion using rhBMP-2. Eur Spine J. 2008 Dec;17(12):1735-44.  **Reason for exclusion:non-RCT** |
| Mesfin A, Buchowski JM, Zebala LP, et al. High-dose rhBMP-2 for adults: major and minor complications: a study of 502 spine cases. J Bone Joint Surg Am. 2013 Sep 4;95(17):1546-53.  **Reason for exclusion:non-RCT** |
| Miyamoto Y, Mabuchi A, Shi D,et al. A functional polymorphism in the 5' UTR of GDF5 is associated with susceptibility to osteoarthritis. Nat Genet. 2007 Apr;39(4):529-33. Epub 2007 Mar 25.  **Reason for exclusion: not about spine** |
| Mobbs RJ, Chung M, Rao PJ. Bone graft substitutes for anterior lumbar interbody fusion. Orthop Surg. 2013 May;5(2):77-85.  **Reason for exclusion:review article** |
| Mödder UI, Clowes JA, Hoey K,et al. Regulation of circulating sclerostin levels by sex steroids in women and in men. J Bone Miner Res. 2011 Jan;26(1):27-34.  **Reason for exclusion: not about spine** |
| Mödder UI, Roforth MM, Hoey K, et al. Effects of estrogen on osteoprogenitor cells and cytokines/bone-regulatory factors in postmenopausal women. Bone. 2011 Aug;49(2):202-7.  **Reason for exclusion:not about spine** |
| Moghaddam-Alvandi A, Zimmermann G, Büchler A, et al. Results of nonunion treatment with bone morphogenetic protein 7 (BMP-7). Unfallchirurg. 2012 Jun;115(6):518-26.  **Reason for exclusion:non-RCT** |
| Mohangi GU, Rothman B, van Zyl AW,et al. Enhanced activity of demineralised bone matrix augmented with xenogeneic bone morphogenetic protein complex in rats. SADJ. 2012 Aug;67(7):354-8.  **Reason for exclusion:animal model** |
| Mroz TE, Wang JC, Hashimoto R,et al. Complications related to osteobiologics use in spine surgery: a systematic review. Spine (Phila Pa 1976). 2010 Apr 20;35(9 Suppl):S86-104.  **Reason for exclusion:review article** |
| Mulconrey DS, Bridwell KH, Flynn J, et al. Bone morphogenetic protein (RhBMP-2) as a substitute for iliac crest bone graft in multilevel adult spinal deformity surgery: minimum two-year evaluation of fusion. Spine (Phila Pa 1976). 2008 Sep 15;33(20):2153-9.  **Reason for exclusion:non-RCT** |
| Mummaneni PV, Pan J, Haid RW,et al. Contribution of recombinant human bone morphogenetic protein-2 to the rapid creation of interbody fusion when used in transforaminal lumbar interbody fusion: a preliminary report. Invited submission from the Joint Section Meeting on Disorders of the Spine and Peripheral Nerves, March 2004. J Neurosurg Spine. 2004 Jul;1(1):19-23.  **Reason for exclusion:non-RCT** |
| Munhoz EA, Ferreira Junior O, Yaedu RY,et al. Radiographic assessment of impacted mandibular third molar sockets filled with composite xenogenic bone graft. Dentomaxillofac Radiol. 2006 Sep;35(5):371-5.  **Reason for exclusion: not about spine** |
| Neen D, Noyes D, Shaw M, et al. Healos and bone marrow aspirate used for lumbar spine fusion: a case controlled study comparing healos with autograft. Spine (Phila Pa 1976). 2006 Aug 15;31(18):E636-40.  **Reason for exclusion:non-RCT** |
| Niehaus AJ, Anderson DE, Samii VF,et al. Effects of orthopedic implants with a polycaprolactone polymer coating containing bone morphogenetic protein-2 on osseointegration in bones of sheep. Am J Vet Res. 2009 Nov;70(11):1416-25.  **Reason for exclusion:animal model.** |
| Neovius E, Lemberger M, Docherty Skogh AC, et al. Alveolar bone healing accompanied by severe swelling in cleft children treated with bone morphogenetic protein-2 delivered by hydrogel. J Plast Reconstr Aesthet Surg. 2013 Jan;66(1):37-42.  **Reason for exclusion:not about spine** |
| Olabisi R. Cell-based therapies for spinal fusion. Adv Exp Med Biol. 2012;760:148-73.  **Reason for exclusion:review article** |
| O'Shaughnessy BA, Kuklo TR, Ondra SL. Surgical treatment of vertebral osteomyelitis with recombinant human bone morphogenetic protein-2. Spine (Phila Pa 1976). 2008 Mar 1;33(5):E132-9  **Reason for exclusion:non-RCT** |
| Ozdemir T, Higgins AM, Brown JL. Osteoinductive biomaterial geometries for bone regenerative engineering. Curr Pharm Des. 2013;19(19):3446-55.  **Reason for exclusion:review article** |
| Padhi D, Jang G, Stouch B,et al. Single-dose, placebo-controlled, randomized study of AMG 785, a sclerostin monoclonal antibody. J Bone Miner Res. 2011 Jan;26(1):19-26.  **Reason for exclusion: not about spine** |
| Papavero L, Zwönitzer R, Burkard I,et al. A composite bone graft substitute for anterior cervical fusion: assessment of osseointegration by quantitative computed tomography. Spine (Phila Pa 1976). 2002 May 15;27(10):1037-43.  **Reason for exclusion: not about lumbar spine** |
| Park JJ, Hershman SH, Kim YH. Updates in the use of bone grafts in the lumbar spine. Bull Hosp Jt Dis (2013). 2013;71(1):39-48.  **Reason for exclusion:review article** |
| Park SB, Park SH, Kim NH,et al. BMP-2 induced early bone formation in spine fusion using rat ovariectomy osteoporosis model. Spine J. 2013 Oct;13(10):1273-80.  **Reason for exclusion: animal model** |
| Pathan E, Abraham S, Van Rossen E, et al. Efficacy and safety of apremilast, an oral phosphodiesterase 4 inhibitor, in ankylosing spondylitis. Ann Rheum Dis. 2013 Sep 1;72(9):1475-80.  **Reason for exclusion:not about spine** |
| Piemonte S, Romagnoli E, Bratengeier C,et al. Serum sclerostin levels decline in post-menopausal women with osteoporosis following treatment with intermittent parathyroid hormone. J Endocrinol Invest. 2012 Oct;35(9):866-8.  **Reason for exclusion:not about spine** |
| Prall WC, Haasters F, Heggebö J,et al. Mesenchymal stem cells from osteoporotic patients feature impaired signal transduction but sustained osteoinduction in response to BMP-2 stimulation. Biochem Biophys Res Commun. 2013 Nov 1;440(4):617-22.  **Reason for exclusion:not about spine** |
| Putzier M, Strube P, Funk J,et al. Periosteal cells compared with autologous cancellous bone in lumbar segmental fusion. J Neurosurg Spine. 2008 Jun;8(6):536-43.  **Reason for exclusion:non-RCT** |
| Ramesh Babu L, Wilson SG, Dick IM,et al. Bone mass effects of a BMP4 gene polymorphism in postmenopausal women. Bone. 2005 Mar;36(3):555-61.  **Reason for exclusion:not about spine** |
| Richmon JD, Sage AB, Shelton E, et al.Effect of growth factors on cell proliferation, matrix deposition, and morphology of human nasal septal chondrocytes cultured in monolayer. Laryngoscope. 2005 Sep;115(9):1553-60.  **Reason for exclusion:not about spine** |
| Riedel GE, Valentin-Opran A. Clinical evaluation of rhBMP-2/ACS in orthopedic trauma: a progress report. Orthopedics. 1999 Jul;22(7):663-5.  **Reason for exclusion: non-RCT** |
| Rihn JA, Kirkpatrick K, Albert TJ. Graft options in posterolateral and posterior interbody lumbar fusion. Spine (Phila Pa 1976). 2010 Aug 1;35(17):1629-39.  **Reason for exclusion:review article** |
| Rihn JA, Gates C, Glassman SD, et al. The use of bone morphogenetic protein in lumbar spine surgery. J Bone Joint Surg Am. 2008 Sep;90(9):2014-25.  **Reason for exclusion:review article** |
| Ristiniemi J, Flinkkilä T, Hyvönen P,et al. RhBMP-7 accelerates the healing in distal tibial fractures treated by external fixation. J Bone Joint Surg Br. 2007 Feb;89(2):265-72.  **Reason for exclusion: not about spine** |
| Rivera JC, Strohbach CA, Wenke JC, Beyond osteogenesis: an in vitro comparison of the potentials of six bone morphogenetic proteins. Front Pharmacol. 2013 Oct 1;4:125.  **Reason for exclusion: not about spine** |
| Rogozinski A, Rogozinski C, Cloud G. Accelerating autograft maturation in instrumented posterolateral lumbar spinal fusions without donor site morbidity. Orthopedics. 2009 Nov;32(11):809.  **Reason for exclusion:non-RCT** |
| Sartori R, Schirwis E, Blaauw B,et al. BMP signaling controls muscle mass. Nat Genet. 2013 Nov;45(11):1309-18.  **Reason for exclusion:review article** |
| Sasso RC, LeHuec JC, Shaffrey C, Iliac crest bone graft donor site pain after anterior lumbar interbody fusion: a prospective patient satisfaction outcome assessment. J Spinal Disord Tech. 2005 Feb;18 Suppl:S77-81.  **Reason for exclusion: different comparison** |
| Schedel H, Schneller A, Vogl T, Dynamic magnetic resonance tomography (MRI): a follow-up study after femur core decompression and instillation of recombinant human bone morphogenetic protein-2 (rhBMP-2) in avascular femur head necrosis Rontgenpraxis. 2000;53(1):16-24.  **Reason for exclusion:not about spine** |
| Schmal H, Niemeyer P, Zwingmann J, et al. Association between expression of the bone morphogenetic proteins 2 and 7 in the repair of circumscribed cartilage lesions with clinical outcome. BMC Musculoskelet Disord. 2010 Jul 29;11:170.  **Reason for exclusion:non-RCT** |
| Schmiedt CW, Lu Y, Heaney K, et al. Comparison of two doses of recombinant human bone morphogenetic protein in absorbable collagen sponges for bone healing in dogs. Am J Vet Res. 2007 Aug;68(8):834-40.  **Reason for exclusion:animal model.** |
| Schmoekel H, Schense JC, Weber FE, Bone healing in the rat and dog with nonglycosylated BMP-2 demonstrating low solubility in fibrin matrices. J Orthop Res. 2004 Mar;22(2):376-81.  **Reason for exclusion:animal model** |
| Shen Y, Liu HX, Ying XZ, et al.Dose-dependent effects of nicotine on proliferation and differentiation of human bone marrow stromal cells and the antagonistic action of vitamin C. J Cell Biochem. 2013 Aug;114(8):1720-8.  **Reason for exclusion:not about spine** |
| Sethi A, Craig J, Bartol S, et al. Radiographic and CT evaluation of recombinant human bone morphogenetic protein-2-assisted spinal interbody fusion. AJR Am J Roentgenol. 2011 Jul;197(1):W128-33.  **Reason for exclusion:non-RCT** |
| Serra E Silva FM, Ricardo de Albergaria-Barbosa J, Mazzonetto R. Clinical evaluation of association of bovine organic osseous matrix and bovine bone morphogenetic protein versus autogenous bone graft in sinus floor augmentation. J Oral Maxillofac Surg. 2006 Jun;64(6):931-5.  **Reason for exclusion: not about spine** |
| Singh K, Dumonski M, Stanley T, et al. Repeat use of human recombinant bone morphogenetic protein-2 for second level lumbar arthrodesis. Spine (Phila Pa 1976). 2011 Feb 1;36(3):192-6.  **Reason for exclusion:animal model.** |
| Slosar PJ, Josey R, Reynolds J. Accelerating lumbar fusions by combining rhBMP-2 with allograft bone: a prospective analysis of interbody fusion rates and clinical outcomes. Spine J. 2007 May-Jun;7(3):301-7. Epub 2007 Jan 2.  **Reason for exclusion:non-RCT** |
| Smadja DM, Bièche I, Silvestre JS,et al. Bone morphogenetic proteins 2 and 4 are selectively expressed by late outgrowth endothelial progenitor cells and promote neoangiogenesis. Arterioscler Thromb Vasc Biol. 2008 Dec;28(12):2137-43.  **Reason for exclusion: not about spine** |
| Smucker JD, Rhee JM, Singh K,et al. Increased swelling complications associated with off-label usage of rhBMP-2 in the anterior cervical spine. Spine (Phila Pa 1976). 2006 Nov 15;31(24):2813-9.  **Reason for exclusion: not about lumbar spine** |
| Southam L, Rodriguez-Lopez J, Wilkins JM,et al. An SNP in the 5'-UTR of GDF5 is associated with osteoarthritis susceptibility in Europeans and with in vivo differences in allelic expression in articular cartilage. Hum Mol Genet. 2007 Sep 15;16(18):2226-32. Epub 2007 Jul 6.  **Reason for exclusion: not about spine** |
| Sproul K, Jones MR, Mathur R, et al. Association study of four key folliculogenesis genes in polycystic ovary syndrome. BJOG. 2010 May;117(6):756-60  **Reason for exclusion: not about spine** |
| Sridharan M, Cheung J, Moore AE, et al. Circulating fibroblast growth factor-23 increases following intermittent parathyroid hormone (1-34) in postmenopausal osteoporosis: association with biomarker of bone formation. Calcif Tissue Int. 2010 Nov;87(5):398-405.  **Reason for exclusion: not about spine** |
| Swiontkowski MF, Aro HT, Donell S,et al. Recombinant human bone morphogenetic protein-2 in open tibial fractures. A subgroup analysis of data combined from two prospective randomized studies. J Bone Joint Surg Am. 2006 Jun;88(6):1258-65.  **Reason for exclusion: not about spine** |
| Tang CL, Zhou Z, Shi WQ. Effects of Fuzheng Huayu Capsule on the ratio of TGF-beta1/BMP-7 of chronic viral hepatitis B fibrosis patients of Gan-Shen insufficiency blood-stasis obstruction syndrome. Zhongguo Zhong Xi Yi Jie He Za Zhi. 2012 Jan;32(1):20-4.  **Reason for exclusion:not about spine** |
| Tang Y, Xie H, Chen J, Activated NF-κB in bone marrow mesenchymal stem cells from systemic lupus erythematosus patients inhibits osteogenic differentiation through downregulating Smad signaling. Stem Cells Dev. 2013 Feb 15;22(4):668-78.  **Reason for exclusion:non-RCT** |
| Teixeira CR, Rahal SC, Volpi RS,et al. Tibial segmental bone defect treated with bone plate and cage filled with either xenogeneic composite or autologous cortical bone graft. An experimental study in sheep. Vet Comp Orthop Traumatol. 2007;20(4):269-76.  **Reason for exclusion:animal model.** |
| Thawani JP, Wang AC, Than KD,et al. Bone morphogenetic proteins and cancer: review of the literature. Neurosurgery. 2010 Feb;66(2):233-46; discussion 246.  **Reason for exclusion:review article** |
| Triplett RG, Nevins M, Marx RE,et al. Pivotal, randomized, parallel evaluation of recombinant human bone morphogenetic protein-2/absorbable collagen sponge and autogenous bone graft for maxillary sinus floor augmentation. J Oral Maxillofac Surg. 2009 Sep;67(9):1947-60.  **Reason for exclusion: not about spine** |
| Tsuzuki N, Otsuka K, Seo J, et al. In vivo osteoinductivity of gelatin β-tri-calcium phosphate sponge and bone morphogenetic protein-2 on an equine third metacarpal bone defect. Res Vet Sci. 2012 Oct;93(2):1021-5.  **Reason for exclusion:not about spine** |
| Vaccaro AR, Patel T, Fischgrund J, A pilot safety and efficacy study of OP-1 putty (rhBMP-7) as an adjunct to iliac crest autograft in posterolateral lumbar fusions. Eur Spine J. 2003 Oct;12(5):495-500.  **Reason for exclusion:non-RCT** |
| Vaccaro AR, Patel T, Fischgrund J,et al. A pilot study evaluating the safety and efficacy of OP-1 Putty (rhBMP-7) as a replacement for iliac crest autograft in posterolateral lumbar arthrodesis for degenerative spondylolisthesis.Spine 2004 29:1885-1892.  **Reason for exclusion:serial report in different follows** |
| Vaccaro AR, Patel T, Fischgrund J, et al.A 2-year follow-up pilot study evaluating the safety and efficacy of op-1 putty (rhbmp-7) as an adjunct to iliac crest autograft in posterolateral lumbar fusions. Eur Spine J. 2005 Sep;14(7):623-9. Epub 2005 Jan 26.  **Reason for exclusion:non-RCT** |
| Vaccaro AR, Whang PG, Patel T, et al.The safety and efficacy of OP-1 (rhBMP-7) as a replacement for iliac crest autograft for posterolateral lumbar arthrodesis: minimum 4-year follow-up of a pilot study.Spine J 2008 8:457-465.  **Reason for exclusion:serial report in different follows** |
| Valdes MA, Thakur NA, Namdari S,et al. Recombinant bone morphogenic protein-2 in orthopaedic surgery: a review. Arch Orthop Trauma Surg. 2009 Dec;129(12):1651-7.  **Reason for exclusion:review article** |
| van den Bergh JP, ten Bruggenkate CM, Groeneveld HH, Recombinant human bone morphogenetic protein-7 in maxillary sinus floor elevation surgery in 3 patients compared to autogenous bone grafts. A clinical pilot study. J Clin Periodontol. 2000 Sep;27(9):627-36.  **Reason for exclusion:not about spine** |
| van Lierop AH, Hamdy NA, van der Meer RW,et al. Distinct effects of pioglitazone and metformin on circulating sclerostin and biochemical markers of bone turnover in men with type 2 diabetes mellitus. Eur J Endocrinol. 2012 Apr;166(4):711-6.  **Reason for exclusion:not about spine** |
| Vaidya R, Sethi A, Bartol S,et al. Complications in the use of rhBMP-2 in PEEK cages for interbody spinal fusions. J Spinal Disord Tech. 2008 Dec;21(8):557-62.  **Reason for exclusion:non-RCT** |
| Vaziri S, Vahabi S, Torshabi M, et al. In vitro assay for osteoinductive activity of different demineralized freeze-dried bone allograft. J Periodontal Implant Sci. 2012 Dec;42(6):224-30.  **Reason for exclusion: not about spine** |
| Verhoeven VJ, Hysi PG, Wojciechowski R, et al.Genome-wide meta-analyses of multiancestry cohorts identify multiple new susceptibility loci for refractive error and myopia. Nat Genet. 2013 Mar;45(3):314-8.  **Reason for exclusion:not about spine** |
| Villavicencio AT, Burneikiene S, Nelson EL,et al. Safety of transforaminal lumbar interbody fusion and intervertebral recombinant human bone morphogenetic protein-2. J Neurosurg Spine. 2005 Dec;3(6):436-43.  **Reason for exclusion:non-RCT** |
| Voskaridou E, Christoulas D, Plata E,et al. High circulating sclerostin is present in patients with thalassemia-associated osteoporosis and correlates with bone mineral density. Horm Metab Res. 2012 Nov;44(12):909-13.  **Reason for exclusion:not about spine** |
| Wang MY, Liu CY. Resorbable polylactic acid interbody spacers with vertebral autograft for anterior cervical discectomy and fusion. Neurosurgery. 2005 Jul;57(1):135-4  **Reason for exclusion:not about lumbar spine** |
| Wilkins RM, Kelly CM, Giusti DE. Bioassayed demineralized bone matrix and calcium sulfate: use in bone-grafting procedures. Ann Chir Gynaecol. 1999;88(3):180-5.  **Reason for exclusion: not about spine** |
| Williams BJ, Smith JS, Fu KM, et al. Does bone morphogenetic protein increase the incidence of perioperative complications in spinal fusion? A comparison of 55,862 cases of spinal fusion with and without bone morphogenetic protein. Spine (Phila Pa 1976). 2011 Sep 15;36(20):1685-91.  **Reason for exclusion:non-RCT** |
| Ye L, Mason MD, Jiang WG. Bone morphogenetic protein and bone metastasis, implication and therapeutic potential. Front Biosci (Landmark Ed). 2011 Jan 1;16:865-97.  **Reason for exclusion:review article** |
| Yuan W, James AW, Asatrian G,et al. NELL-1 based demineralized bone graft promotes rat spine fusion as compared to commercially available BMP-2 product. J Orthop Sci. 2013 Jul;18(4):646-57. doi: 10.1007/s00776-013-0390-5. Epub 2013 May 18  **Reason for exclusion:animal model.** |
| Zhou YJ, Li A, Song YL,et al. Role of sclerostin in the bone loss of postmenopausal chinese women with type 2 diabetes. Chin Med Sci J. 2013 Sep;28(3):135-9.  **Reason for exclusion:not about spine** |
| Zhou N, Wang J, Liu C,et al. Clinical application of bioactive CPC loading rhBMP-2 in repairing bone defects. Zhongguo Xiu Fu Chong Jian Wai Ke Za Zhi. 2009 Mar;23(3):257-60.  **Reason for exclusion: not about spine** |
| Zimmermann G, Moghaddam A, Wagner C,et al. Clinical experience with bone morphogenetic protein 7 (BMP 7) in nonunions of long bones. Unfallchirurg. 2006 Jul;109(7):528-37.  **Reason for exclusion: not about spine** |

List of excluded studies (Articles selected in the first round, where disagreement exists)

| Sasso RC, LeHuec JC, Shaffrey C; Spine Interbody Research Group. Iliac crest bone graft donor site pain after anterior lumbar interbody fusion: a prospective patient satisfaction outcome assessment.J Spinal Disord Tech. 2005 Feb;18 Suppl:S77-81.  **Reason for exclusion:non-RCT** |
| --- |
| Singh K, Dumonski M, Stanley T, et al. Repeat use of human recombinant bone morphogenetic protein-2 for second level lumbar arthrodesis.Spine (Phila Pa 1976). 2011 Feb 1;36(3):192-6. doi: 10.1097/BRS.0b013e3181cdd396.  **Reason for exclusion:animal model** |
| Carragee EJ, Chu G, Rohatgi R, et al.Cancer risk after use of recombinant bone morphogenetic protein-2 for spinal arthrodesis.J Bone Joint Surg Am. 2013 Sep 4;95(17):1537-45. doi: 10.2106/JBJS.L.01483.  **Reason for exclusion:non-RCT** |
| [Pimenta L](http://www.ncbi.nlm.nih.gov/pubmed?term=Pimenta L[Author]&cauthor=true&cauthor_uid=23444134), [Marchi L](http://www.ncbi.nlm.nih.gov/pubmed?term=Marchi L[Author]&cauthor=true&cauthor_uid=23444134), [Oliveira L](http://www.ncbi.nlm.nih.gov/pubmed?term=Oliveira L[Author]&cauthor=true&cauthor_uid=23444134), et al. [A Prospective, Randomized, Controlled Trial Comparing Radiographic and Clinical Outcomes between Stand-Alone Lateral Interbody Lumbar Fusion with either Silicate Calcium Phosphate or rh-BMP2.](http://www.ncbi.nlm.nih.gov/pubmed/23444134)[J Neurol Surg A Cent Eur Neurosurg.](http://www.ncbi.nlm.nih.gov/pubmed) 2013 Nov;74(6):343-50. doi: 10.1055/s-0032-1333420.  **Reason for exclusion:different comparison** |
| Burkus JK, Gornet MF, Glassman SD, et al. Blood serum antibody analysis and long-term follow-up of patients treated with recombinant human bone morphogenetic protein-2 in the lumbar spine.Spine (Phila Pa 1976). 2011 Dec1;36(25):2158-67. doi: 10.1097/BRS.0b013e3182059a8c.  **Reason for exclusion:non-RCT** |
| Sethi A, Craig J, Bartol S, Chen W, et al.Radiographic and CT evaluation of recombinant human bone morphogenetic protein-2-assisted spinal interbody fusion.AJR Am J Roentgenol. 2011 Jul;197(1):W128-33. doi: 10.2214/AJR.10.5484.  **Reason for exclusion:non-RCT** |
| Vaccaro AR, Patel T, Fischgrund J, et al. A 2-year follow-up pilot study evaluating the safety and efficacy of op-1 putty (rhbmp-7) as an adjunct to iliac crest autograft in posterolateral lumbar fusions.Eur Spine J. 2005 Sep;14(7):623-9. Epub 2005 Jan 26.  **Reason for exclusion:non-RCT** |
| Mummaneni PV, Pan J, Haid RW, et al.Contribution of recombinant human bone morphogenetic protein-2 to the rapid creation of interbody fusion when used in transforaminal lumbar interbody fusion: a preliminary report. Invited submission from the Joint Section Meeting on Disorders of the Spine and Peripheral Nerves, March 2004.J Neurosurg Spine. 2004 Jul;1(1):19-23.  **Reason for exclusion:non-RCT** |
